# Apigenin Derivatives Alleviate OVA-Induced Oxidative Stress in Bronchial Asthma: A Structure-Activity Relationship Study

**DOI:** 10.3390/molecules31162768

**Published:** 2026-08-09

**Authors:** Chenliang Li, Lijin Xiao, Wei Wu, Lingyang Kong, Shuyuan Yue, Zhijie Zhan, Jiao Xu, Wei Ma

**Affiliations:** 1 School of Pharmacy, Heilongjiang University of Chinese Medicine, Harbin 150040, China; l7174080625@163.com (C.L.); 15260756338@163.com (L.X.); wuwei52414@163.com (W.W.); hljkly970219@163.com (L.K.); 15639358645@163.com (S.Y.); zzj747664041@163.com (Z.Z.); 2 Jiamusi College, Heilongjiang University of Chinese Medicine, Jiamusi 154007, China; 3 Key Laboratory of Northern Medicine Basis and Application, Ministry of Education, Heilongjiang University of Chinese Medicine, Harbin 150040, China

**Keywords:** apigenin, chemical synthesis, bronchial asthma, oxidative stress, structure-activity relationship

## Abstract

Apigenin (API) is a flavonoid compound widely distributed in nature. The global prevalence of asthma is increasing year by year, influenced by various factors and difficult to cure completely, and new drugs and therapies are constantly emerging. Although API is a low-toxicity flavonoid compound, its poor water solubility and low bioavailability present limitations in the treatment of asthma. In this study, the structure of API was chemically modified by introducing acyl and alkyl groups while preserving its original structure. The structures were identified using FT-IR, ^1^H-NMR and ^13^C-NMR spectroscopy, yielding derivatives (A–J). To further investigate the effects of structural modifications on API’s biological activity, an ovalbumin (OVA)-induced asthma model was established in mice to evaluate the antioxidants’ activity. Hematoxylin-eosin staining was used to observe pathological changes in lung tissue, and oxidative stress-related parameters, including ROS, SOD, and MDA, were measured to assess the derivatives’ protective effects against oxidative damage. The results showed that the 10 synthetic derivatives exhibited varying degrees of oxidative stress during treatment. Compared with the model group, the API derivative treatment group significantly reduced ROS and MDA levels and increased SOD activity. Moreover, treatment with the compounds reduced the levels of pro-inflammatory cytokines, including TNF-α, IL-6, and IL-1β, and decreased serum IgE levels. Histopathological examination further demonstrated that the compounds alleviated inflammatory cell infiltration and tissue damage in the lungs. Structure-activity analysis indicated that, among the 10 derivatives, the tri-substituted API derivatives exhibited superior antioxidant activity compared to the di-substituted API derivatives. By modifying the chemical structure of API, its antioxidant activity in OVA-induced bronchial asthma was significantly enhanced. 5,7,4′-O-triethyl API and 5,7,4′-O-triacetyl API demonstrated therapeutic effects comparable to those of dexamethasone and show promise as lead compounds for the development of novel asthma treatments.

## 1. Introduction

Bronchial asthma is a common chronic inflammatory airway disease primarily characterized by airway hyperresponsiveness and reversible airflow limitation. The causes of bronchial asthma are numerous and are influenced by genetic and environmental factors. Common types of bronchial asthma include exercise-induced, drug-induced, occupational, and allergic asthma [1]. In recent years, with the worsening of environmental pollution and increased exposure to allergens, the incidence of bronchial asthma has shown a steady upward trend, becoming a major public health issue affecting human health. Patients typically experience bronchial asthma attacks in the early morning and late at night; for most patients, these attacks resolve on their own or with treatment [2]. Currently, common medications for treating bronchial asthma include inhaled corticosteroids and β2-agonists. Although these treatments can effectively relieve clinical symptoms, long-term use can lead to drug resistance and adverse reactions, and they are unable to fundamentally halt the progression of the disease [3]. Therefore, the development of safe and effective new anti-asthma drugs remains of great importance. Numerous studies have shown that oxidative stress plays a key role in the pathogenesis of bronchial asthma [4]. During an asthma attack, inflammatory cells such as eosinophils, neutrophils, and macrophages accumulate in large numbers in the airways, releasing excessive amounts of ROS, which leads to oxidative stress [5,6]. Furthermore, during an asthma attack, airway remodeling and hyperresponsiveness lead to inflammation. In the process of oxidative stress, excessive ROS can trigger lipid peroxidation, oxidative damage to proteins, and DNA damage, resulting in impaired airway epithelial barrier function, which in turn further promotes the release of inflammatory cytokines, airway remodeling, and the development of airway hyperresponsiveness [7,8]. Studies have shown that chronic inflammation and oxidative damage to airway epithelial cells increase the production of oxidants, leading to a series of pathophysiological changes, including tissue damage, exacerbated airway inflammation, and enhanced airway hyperresponsiveness [9].

**Figure 1:**
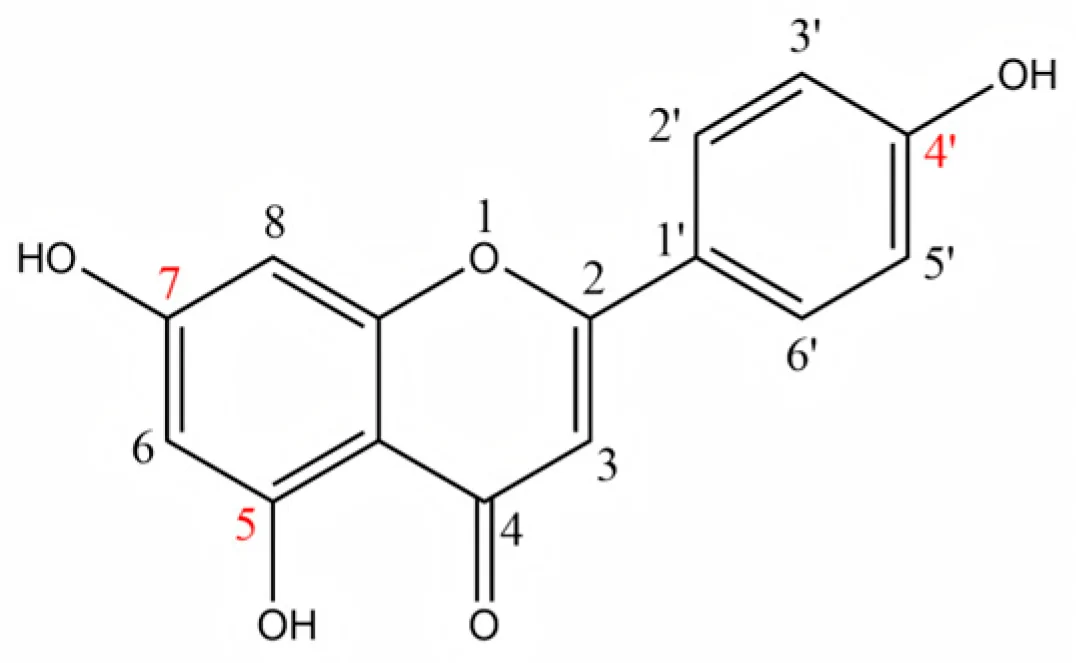
Structural formula of apigenin.

In recent years, natural extracts and compounds with health benefits have attracted significant attention. Flavonoids, as one of the substances effective in the prevention and treatment of bronchial asthma, can improve airway inflammation and airway remodeling through various mechanisms, thereby reducing airway hyperresponsiveness and achieving therapeutic effects [10]. Apigenin (4′,5,7-trihydroxyflavone, API, See Figure 1), as one of the typical flavonoid compounds widely distributed in natural plants, offers advantages such as low toxicity and easy availability. Studies have demonstrated that several types of apigenin derivatives have been synthesized, including O-alkylation, O-acylation, esterification, etherification, glycosylation, and conjugation with various pharmacophores, which yield derivatives with improved pharmacological activities and physicochemical properties [11]. Modern pharmacological studies have shown that API possesses anti-inflammatory, antioxidant, and antitumor properties, as well as efficacy in treating cardiovascular diseases [12,13,14]. Research has shown that API can increase the levels of AMPK, Nrf2, and HO-1 in mouse lung tissue and has an inhibitory effect on airway inflammation that occurs during bronchial asthma attacks [15]. In addition to respiratory diseases, the protective effects of apigenin have also been demonstrated in liver, kidney, cardiovascular, and neurological injury models, where it alleviates inflammation, oxidative stress, and tissue damage through modulation of NF-κB, Nrf2/HO-1, and MAPK signaling pathways [16]. Similar biological activities have also been reported for structurally related flavonoids such as luteolin, suggesting that flavone derivatives may represent promising therapeutic agents for inflammatory disorders. However, the presence of multiple phenolic hydroxyl groups in the structure of API results in poor lipophilicity, low bioavailability, and rapid metabolism in the body, thereby limiting its further development and application [17]. Structural modification is one of the key strategies for improving the pharmacodynamics and pharmacokinetics of natural products; targeted structural modifications to API can enhance its lipophilicity and bioavailability [18].

Due to the poor lipophilicity of API, we used API as a lead compound in our study and designed and synthesized a series of API derivatives via alkylation and acylation. In the present study, structural modification of apigenin was performed to explore the structure-activity relationships of its derivatives and to evaluate whether these modifications could enhance their biological activities compared with the parent compound. We characterized the structures of 10 derivatives using Fourier-transform infrared spectroscopy (FT-IR), nuclear magnetic resonance hydrogen spectroscopy (^1^H-NMR) and Carbon-13 nuclear magnetic resonance spectroscopy (^13^C-NMR). By establishing an OVA-induced bronchial asthma mouse model, we systematically evaluated the antioxidant activity of each derivative by measuring oxidative stress markers such as reactive oxygen species (ROS), malondialdehyde (MDA), and superoxide dismutase (SOD), combined with pulmonary histopathological observations. We further explored their preliminary structure-activity relationships, providing experimental evidence and theoretical references for the development of API-based anti-asthma drug candidates.

## 2. Results

### 2.1. Results of the Synthesis of Alkylated Derivatives of Apigenin

**Figure 2:**
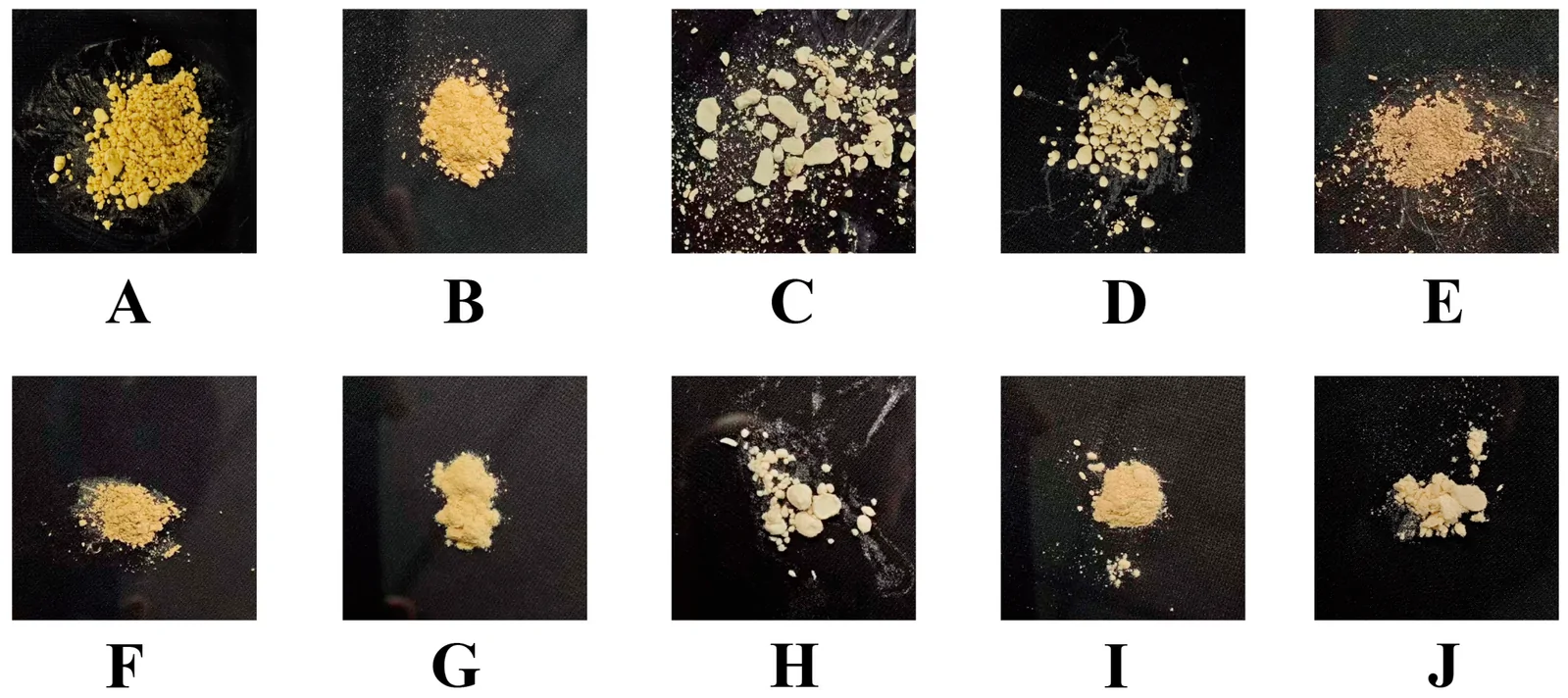
Sample photographs of synthetic derivatives (**A**–**J**).

Based on ‘3.2. Synthesis of Alkylated Derivatives of Apigenin’ and ‘3.4. Structural Characterization of Derivatives A–J’, we successfully synthesized di-substituted and tri-substituted alkylated derivatives of API (A-D) and characterized their structures using FT-IR, 1H-NMR and ^13^C NMR spectroscopy. See Figure 2A–D.

7,4′-O-Dimethyl-apigenin (A): white solid, yield approximately 72.84%. FT-IR (KBr) v: 3753.60, 2843.17, 1670.41, 1246.06, 1091.75, 817.85. ^1^H NMR (400 MHz, DMSO-d_6_): δ 12.89 (s, 1H), 8.03 (d, J = 8.9 Hz, 2H), 7.10 (d, J = 8.9 Hz, 2H), 6.88 (s, 1H), 6.76 (d, J = 2.3 Hz, 1H), 6.35 (d, J = 2.2 Hz, 1H), 3.85 (d, J = 2.9 Hz, 6H, 2×CH_3_). ^13^C NMR (101 MHz, DMSO-d_6_): δ 182.69, 168.91, 165.25, 162.23, 161.20, 156.53, 156.04, 129.12, 128.78, 121.06, 116.54, 108.38, 105.58, 103.71, 101.85, 21.54, 21.36. The FT-IR spectrum shows a phenolic hydroxyl absorption at 3753.60 cm^−1^, confirming the retention of the C-5 hydroxyl group, while peaks at 1246.06 cm^−1^ and 1091.75 cm^−1^ indicate successful methylation at the 7- and 4′-positions. in the ^1^H-NMR spectrum, δ 12.89 corresponds to the signal from the active hydrogen of the C-5 hydroxyl group, and δ 3.85 corresponds to the characteristic hydrogen signals of the two methoxy groups. The molecular formula of this compound is confirmed to be C_19_H_14_O_7_. Combined with the IR spectral data, the structure of this compound can be identified as 7,4′-O-dimethylapigenin, which is consistent with the target structure.

7,4′-O-Diethyl-apigenin (B): white solid, yield 74.84%. FT-IR (KBr) v: 3753.60, 2926.05, 2881.75, 1269.20, 1037.74, 810.13. ^1^H-NMR (400 MHz, DMSO-d_6_): δ 12.88 (s, 1H, 5-OH), 8.03 (d, J = 8.8 Hz, 2H, H-2′, H-6′), 7.09 (d, J = 8.8 Hz, 2H, H-3′, H-5′), 6.88 (s, 1H, H-3), 6.76 (br s, 2H, H-6 and H-8), 4.14 (q, J = 6.9 Hz, 4H, 2×OCH_2_), 1.35 (t, J = 6.9 Hz, 6H, 2×CH_3_). ^13^C NMR (101 MHz, DMSO-d_6_): δ 183.05, 169.38, 168.92, 164.02, 161.22, 156.75, 156.38, 154.01, 128.69, 128.39, 123.21, 108.67, 106.21, 105.90, 102.18, 21.39, 21.37. The phenolic hydroxyl absorption peak at 3753.60 cm^−1^ in the FT-IR spectrum confirms the retention of the C-5 hydroxyl group, demonstrating the successful ethylation of the 7- and 4′-position phenolic hydroxyl groups; In the ^1^H-NMR spectrum, the signal at δ 12.88 corresponds to the active hydrogen of the hydroxyl group at the C-5 position, while the signals at δ 4.14 and δ 1.35 correspond to the characteristic hydrogen signals of the two ethoxy groups. The molecular formula of this compound is confirmed to be C_21_H_16_O_7_. These data corroborate one another, confirming that the structure of this compound is 7,4′-O-diethylapigenin, which is consistent with the target structure.

5,7,4′-O-Trimethyl-apigenin (C): white solid, yield 75.08%. FT-IR (KBr) v: 2980, 2840, 1250, 1080, 815. ^1^H-NMR (400 MHz, DMSO-d_6_): δ 8.05 (d, J = 8.9 Hz, 2H), 7.11 (d, J = 9.0 Hz, 2H), 6.91 (s, 1H), 6.78 (d, J = 2.3 Hz, 1H), 6.37 (d, J = 2.2 Hz, 1H), 3.86 (t, J = 2.9 Hz, 9H, 3×CH_3_). ^13^C NMR (101 MHz, DMSO-d_6_): δ 183.00, 172.67, 172.27, 161.18, 156.92, 152.80, 151.09, 129.17, 128.60, 123.10, 116.46, 108.45, 106.08, 105.59, 103.75, 101.85, 27.39, 24.30. In the FT-IR spectrum, the region from 2980 to 2840 cm^−1^ corresponds to the characteristic -OCH_3_ saturated alkyl group, while the region from 1250 to 1080 cm^−1^ corresponds to the core peak of the ether bond, indicating complete methylation at the 5-, 7-, and 4′-positions. In the 1H-NMR spectrum, the δ value of 3.86 corresponds to the characteristic signal of a trimethoxy group, and the absence of a C-5 reactive hydrogen signal is highly consistent with the structure of 5,7,4′-O-trimethyl apigenin. The molecular formula of this compound is confirmed to be C_21_H_18_O_7_.

5,7,4′-O-Triethyl-apigenin (D): white solid, yield 71.78%. FT-IR (KBr) v: 2997.48, 1742.51, 1654.42, 1508.38, 1265.35, 898.86. ^1^H-NMR (400 MHz, DMSO-d_6_): δ 8.03 (d, J = 8.8 Hz, 2H), 7.09 (d, J = 8.8 Hz, 2H), 6.88 (s, 1H), 6.76 (s, 2H), 6.34 (s, 1H), 4.14 (p, J = 6.5 Hz, 4H, 2×CH_2_), 1.35 (t, J = 6.9 Hz, 9H, 3×CH_3_). ^13^C NMR (126 MHz, DMSO-d_6_): δ 183.05, 172.76, 172.32, 163.99, 161.24, 156.76, 156.47, 154.08, 128.67, 128.34, 123.18, 108.64, 106.17, 105.86, 102.16, 27.46, 27.42, 9.23, 9.18. The FT-IR spectrum shows no absorption from free phenolic hydroxyl groups, and the ^1^H-NMR spectrum lacks a C-5 hydrogen signal; furthermore, the δ value of 4.14 corresponds to three sets of characteristic ethoxy hydrogen signals, confirming that the structure matches the target. The molecular formula of this compound is confirmed to be C_24_H_22_O_8_.

### 2.2. Results of the Synthesis of Acetylated Derivatives of Apigenin

Based on ‘3.3. Synthesis of acylated apigenin derivatives’ and ‘3.4. Structural Characterization of Derivatives A–J’, we successfully synthesized six acylated API derivatives and characterized their structures using FT-IR, 1H-NMR and ^13^C NMR spectroscopy. See Figure 2E–J.

7,4′-O-Diacetyl-apigenin (E): A yellow solid product, with a yield of 68.16%. FT-IR (KBr) v: 3753.60, 2978., 1778.43, 1654.98, 1246.06, 837.13. ^1^H-NMR (400 MHz, DMSO-d_6_): δ 13.01 (s, 1H), 7.97 (d, J = 8.9 Hz, 2H), 7.05 (d, J = 8.8 Hz, 2H), 6.95 (d, J = 2.1 Hz, 1H), 6.97 (s, 1H), 6.63 (d, J = 2.1 Hz, 1H), 2.31 (s, 6H, 2×CH_3_). ^13^C NMR (101 MHz, DMSO-d_6_): δ 183.01, 171.83, 171.39, 163.95, 161.24, 156.73, 156.36, 153.99, 128.66, 128.33, 123.14, 108.62, 106.13, 105.81, 102.13, 35.77, 18.25, 18.21, 13.83, 13.80. In the FT-IR spectrum, the broad characteristic hydroxyl peak at 3753.60 cm^−1^ appeared simultaneously with the characteristic ester carbonyl peak at 1751.42 cm^−1^, confirming the retention of the 5-hydroxyl group and the successful acetylation of the 7- and 4′-hydroxyl groups. The appearance of the characteristic C-5 hydrogen signal at δ 13.01 in the ^1^H-NMR spectrum confirms that the 5-hydroxyl group has not undergone acylation, while the characteristic single peak of the acetyl methyl group at δ 2.31 further confirms the successful acylation of the 7- and 4′-hydroxyl groups. The molecular formula of this compound is confirmed to be C_23_H_22_O_7_. These data corroborate one another, further confirming the correct structure of the target compound.

7,4′-O-Dipropionyl-apigenin (F): A yellow solid product, with a yield of 68.34%. FT-IR (KBr): v: 3753.60, 2985, 1759.14, 1654.98, 1296, 1495, 895. ^1^H-NMR (DMSO-d_6_, 400 MHz): δ 12.83 (s, 1H), 8.17 (d, J = 8.9 Hz, 2H), 7.37 (d, J = 8.9 Hz, 2H), 7.14 (s, 1H), 7.11 (d, J = 2.0 Hz, 1H), 6.67 (d, J = 2.1 Hz, 1H), 2.70–2.60 (m, 2×CH_2_, 4H), 1.15 (t, J = 7.5 Hz, 2×CH_3_, 6H). ^13^C NMR (101 MHz, DMSO-d_6_): δ 183.00, 171.83, 171.39, 163.95, 161.24, 156.72, 156.36, 153.99, 128.65, 128.32, 123.13, 108.62, 106.12, 105.81, 102.13, 35.77, 18.25, 18.21, 13.83, 13.79. The above hydrogen spectrum characteristics match the structure of 7,4′-O-dipropionyl apigenin; the presence of the characteristic C-5 hydrogen signal at δ 12.83 indicates that the 5-hydroxyl group has not undergone acylation, while the propionyl α-methylene peak at δ 2.70–2.60 and the terminal methyl peak at δ 1.15 further confirm the successful acylation of the 7- and 4′-hydroxyl groups. The molecular formula of this compound is confirmed to be C_27_H_28_O_8_.

7,4′-O-Dibutyryl-apigenin (G): yellow solid product, yield 69%. FT-IR (KBr) v: 3753.60, 3078.49, 2935.76, 1658.84, 1589.40, 1091.75. ^1^H-NMR (DMSO-d_6_, 400 MHz): 12.83 (s, 1H), 8.17 (d, J = 8.9 Hz, 2H), 7.36 (d, J = 8.8 Hz, 2H), 7.14 (s, 1H), 7.10 (d, J = 2.0 Hz, 1H), 6.66 (d, J = 2.0 Hz, 1H), 2.60 (t, J = 7.3 Hz, 2×-COCH_2_, 4H), 1.68 (m, J = 7.4 Hz, 2×-CH_2_CH_3_, 4H), 0.99 (t, J = 7.4 Hz, 3×-CH_3_, 6H). ^13^C NMR (101 MHz, DMSO-d_6_): δ 182.44, 165.66, 164.10, 162.89, 161.65, 161.64, 157.72, 128.85, 123.15, 115.06, 105.18, 104.15, 98.51, 93.20, 56.54, 56.05. The above hydrogen spectrum features match the structure of 7,4′-O-dibutyryl apigenin, with the 5-OH group retained and butyryl substitutions at the 7- and 4′-positions. The appearance of a broad, characteristic intramolecular hydrogen-bonding peak at δ 12.8–12.9 for the 5-OH group confirms that the 5-hydroxyl group has not undergone acylation, thereby confirming its structure. In the FT-IR spectrum, the characteristic broad hydroxyl peak at 3432 cm^−1^ appeared simultaneously with the characteristic ester carbonyl peak at 1736 cm^−1^, supporting the retention of the 5-hydroxyl group and the successful butyrylation of the 7- and 4′-hydroxyl groups. This finding is consistent with the ^1^H-NMR data and further confirms the correct structure of the target compound. The molecular formula of this compound is confirmed to be C_17_H_14_O_5_.

5,7,4′-O-Triacetyl-apigenin (H): A yellow solid product, with a yield of 74.75%. FT-IR (KBr) v: 2982.05, 1633.41, 1562.39, 1427.37, 1261.49, 1037.74. ^1^H-NMR (400 MHz, DMSO-d_6_): δ 8.16 (d, J = 8.9 Hz, 2H), 7.37 (d, J = 8.8 Hz, 2H), 7.10 (d, J = 2.0 Hz, 1H), 7.13 (s, 1H), 6.67 (d, J = 2.0 Hz, 1H), 2.31 (s, 3×CH_3_, 9H). ^13^C NMR (126 MHz, DMSO-d_6_): δ 182.44, 165.66, 164.11, 162.89, 161.65, 157.73, 128.87, 123.15, 115.06, 105.20, 104.16, 98.50, 93.20, 56.55, 56.05. The above hydrogen spectrum characteristics match the structure of 7,4′,5-O-triacetylapigenin. The absence of a characteristic hydrogen signal at C-5 (δ 12.5–13.2) confirms that the hydroxyl group at the 5-position has been successfully acetylated, and the three characteristic single peaks of acetyl methyl groups at δ 2.31 further confirm that the hydroxyl groups at the 5-, 7-, and 4′-positions have all been acetylated, thereby confirming its structure. The molecular formula of this compound is confirmed to be C_18_H_16_O_5_.

5,7,4′-O-Tripropionyl-apigenin (I): A yellow solid product, with a yield of 74.54%. FT-IR (KBr) v: 2985.91, 1759.14, 1654.98, 1508.38, 1265.35, 895.00. ^1^H-NMR (DMSO-d_6_, 400 MHz): δ 8.17 (d, J = 8.9 Hz, 2H), 7.37 (d, J = 8.9 Hz, 2H), 7.14 (s, 1H), 7.11 (d, J = 2.1 Hz, 1H), 6.68 (d, J = 2.1 Hz, 1H), 2.65 (q, J = 7.5 Hz, 3×CH_2_, 6H), 1.15 (t, J = 7.5 Hz, 3×CH_3_, 9H). ^13^C NMR (126 MHz, DMSO-d_6_): δ 182.42, 164.94, 164.11, 162.19, 161.65, 157.77, 128.88, 123.00, 116.45, 115.44, 105.12, 104.09, 98.79, 93.52, 64.67, 64.08, 14.87. The above hydrogen spectrum characteristics match the structure of 7,4′,5-O-triacetylapigenin, and the absence of a broad, characteristic C-5 hydrogen peak at δ 12.83 confirms that the 5-hydroxyl group has been successfully acylated. The propionyl α-methylene peak at δ 2.65 and the terminal methyl peak at δ 1.15 further confirm that the 5-, 7-, and 4′-hydroxyl groups have all been acylated, thereby confirming the structure. The molecular formula of this compound is confirmed to be C_19_H_18_O_5_.

5,7,4′-O-Tributyryl-apigenin (J): A yellow solid, isolated in 68.49% yield. FT-IR (KBr) v: 3078.49, 2935.76, 1658.84, 1589.40, 1492.95, 1091.75. ^1^H-NMR (DMSO-d_6_, 400 MHz): δ 8.18 (d, J = 8.8 Hz, 2H), 7.36 (d, J = 8.8 Hz, 2H), 7.14 (s, 1H), 7.11 (d, J = 2.0 Hz, 1H), 6.66 (d, J = 2.1 Hz, 1H), 2.61 (t, J = 7.0 Hz, 3×-COCH_2_, 6H), 1.69 (m, J = 7.3 Hz, 3×-CH_2_CH_3_, 6H), 0.99 (t, J = 7.4 Hz, 3×-CH_3_, 9H). ^13^C NMR (126 MHz, DMSO-d_6_): δ 182.40, 164.92, 164.07, 163.24, 161.64, 161.50, 159.60, 157.73, 128.85, 123.34, 115.41, 115.28, 107.14, 104.06, 97.70, 93.51, 64.81, 64.66, 64.45, 31.17, 14.99, 14.96, 14.89, 14.86. The above hydrogen spectrum features match the structure of 7,4′-O-dibutyryl apigenin, with the 5-OH group retained and butyryl substitutions at the 7- and 4′-positions. The absence of a broad single peak at δ 12.8–12.9, characteristic of intramolecular hydrogen bonding of the 5-OH group, confirms that the 5-hydroxyl group has not undergone acylation. Combined with the IR spectrum, this confirms its structure. The molecular formula of this compound is confirmed to be C_21_H_22_O_5_.

### 2.3. Effects on Body Weight in Mice with an OVA-Induced Asthma Model

**Figure 3:**
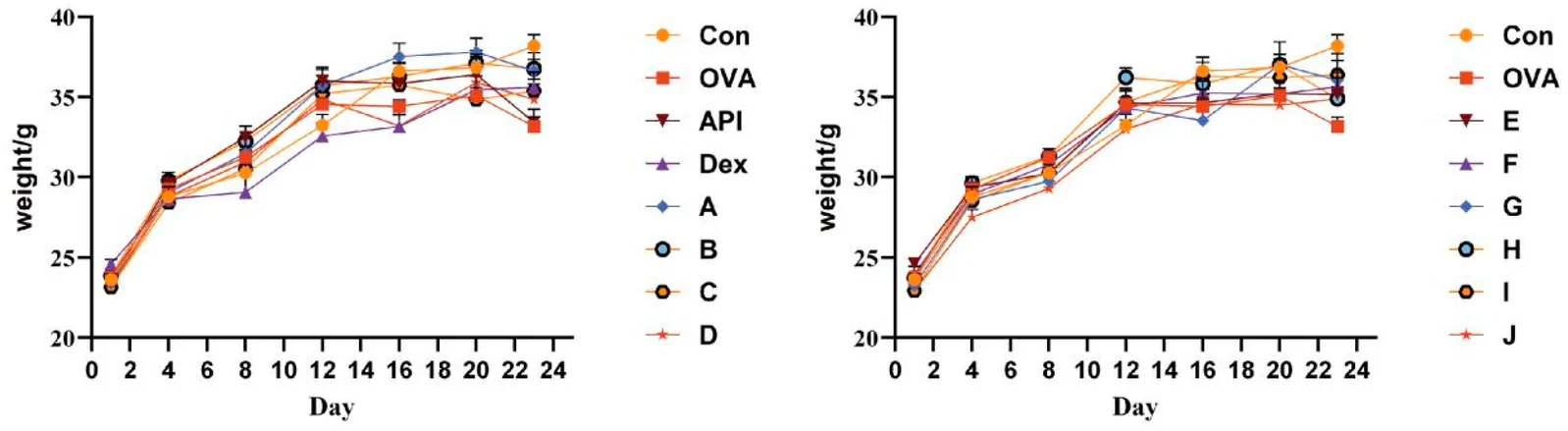
Trends in Body Weight Changes Among the Different Groups of Mice Over 24 Days. Compared with the Con group, the weight gain rate of the OVA model group was significantly decreased (*p* < 0.01). API and all apigenin derivatives (A–J) remarkably reversed OVA-induced growth retardation (*p* < 0.05). Among all derivatives, tri-substituted alkylated compounds A and C showed weight gain close to that of the Con group, while B, D and acylated derivatives E–J exhibited an improvement effect similar to that of parent API; the overall body weight recovery in all modified derivative-treated groups was less pronounced than that in the positive control Dex group. Body weight changes during the experimental period. Data are presented as mean ± SD (*n* = 8 per group).

During the experiment, body weight changes in 112 female mice were dynamically monitored from days 0 to 22 to evaluate the safety and potential therapeutic effects of API and its derivatives at a dose of 20 mg/kg in an OVA-induced asthma model. The results showed that body weight in all groups exhibited a consistent and stable upward trend, with no sudden drops or growth stagnation observed, indicating good drug safety and overall tolerability in the animals. Mice in the Con group consistently exhibited the highest rate of weight gain, reflecting a normal physiological state. Mice in the OVA group showed a significantly slower rate of weight gain compared to the Con group, indicating that the OVA model was successfully established and providing a reliable pathological basis for subsequent efficacy evaluations. Mice in the Dex and API groups had slightly higher body weights than those in the OVA group but lower than those in the Con group. Among the 10 apigenin derivative groups, Groups A, C, and F exhibited weight gain rates close to those of the Con group, effectively alleviating growth inhibition. The body weight gain rates in Groups B, D, E, G, H, I, and J were similar to those in the API group, all of which improved the growth inhibition observed in the OVA model. The results are shown in Figure 3.

### 2.4. Effects on the Lungs and Spleens of Mice in the OVA Asthma Model

**Figure 4:**
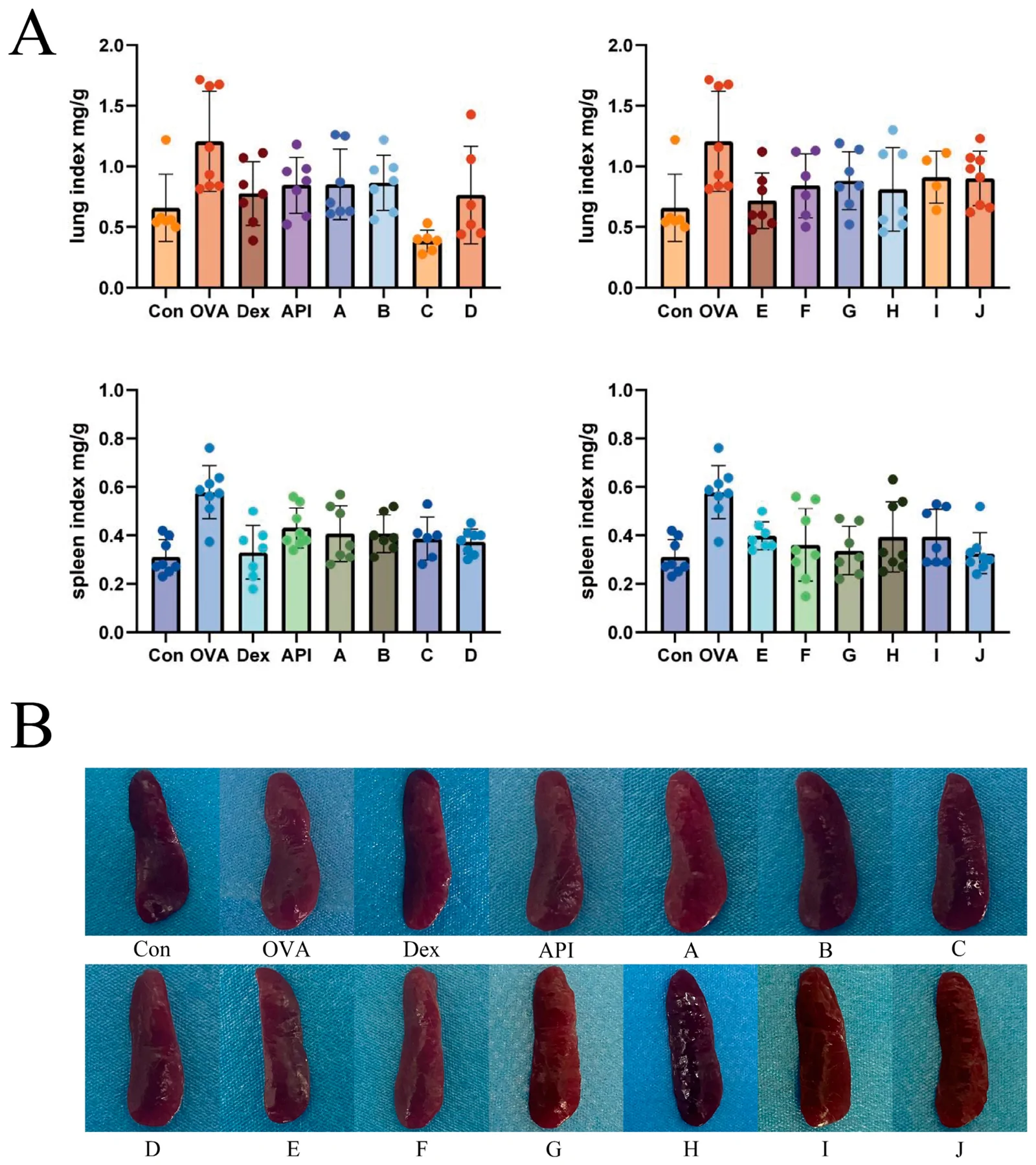
Lung and spleen indices and spleen images in each group of the OVA-induced mouse asthma model ((**A**) lung and spleen indices; (**B**) spleen images). Compared with the Con group, OVA treatment significantly elevated the lung index and spleen index (*p* < 0.01). Dex and API markedly reduced two organ indexes relative to the OVA group (*p* < 0.01, *p* < 0.05, respectively). Most derivatives exerted stronger anti-inflammatory and immunoregulatory effects than raw API (*p* < 0.05), among which alkylated B, alkylated D, acylated F, acylated H displayed the most significant reductions on lung and spleen indices (*p* < 0.01), but their efficacy was still weaker than that of Dex. Tri-substituted derivatives exhibited better improvement than di-substituted analogs on immune organ abnormality. Data are presented as mean ± SD. Error bars represent the standard deviation, and each dot represents one technical replicate (*n* = 3).

The lung index and spleen index can serve as auxiliary indicators for evaluating pulmonary inflammation and changes in immune organs [19,20]. As shown in Figure 4, compared with the Con group, both the lung index and spleen index were significantly elevated in the OVA group (*p* < 0.01), suggesting that OVA sensitization and challenge successfully induced pulmonary inflammation and changes in immune organs, indicating that the asthma model was successfully established. Compared with the OVA group, the Dex group significantly reduced both the lung index and spleen index (*p* < 0.01), and the API group also significantly improved both indices (*p* < 0.05), indicating that both groups possess certain anti-inflammatory and immunomodulatory effects. Compared with the API group, most apigenin derivatives showed a better trend of improvement, among which alkylated derivative groups B and D, as well as acetylated derivative groups F and H, exhibited more significant effects (*p* < 0.01); however, the overall improvement was still weaker than that of the Dex group. The above results indicate that structural modifications of API can further enhance its ability to alleviate OVA- induced pulmonary inflammation and immune organ abnormalities, with some alkylated and acetylated derivatives exhibiting good in vivo anti-inflammatory activity.

### 2.5. Effects on Inflammatory Cytokines in the Lavage Fluid of Asthma Model Mice

**Figure 5:**
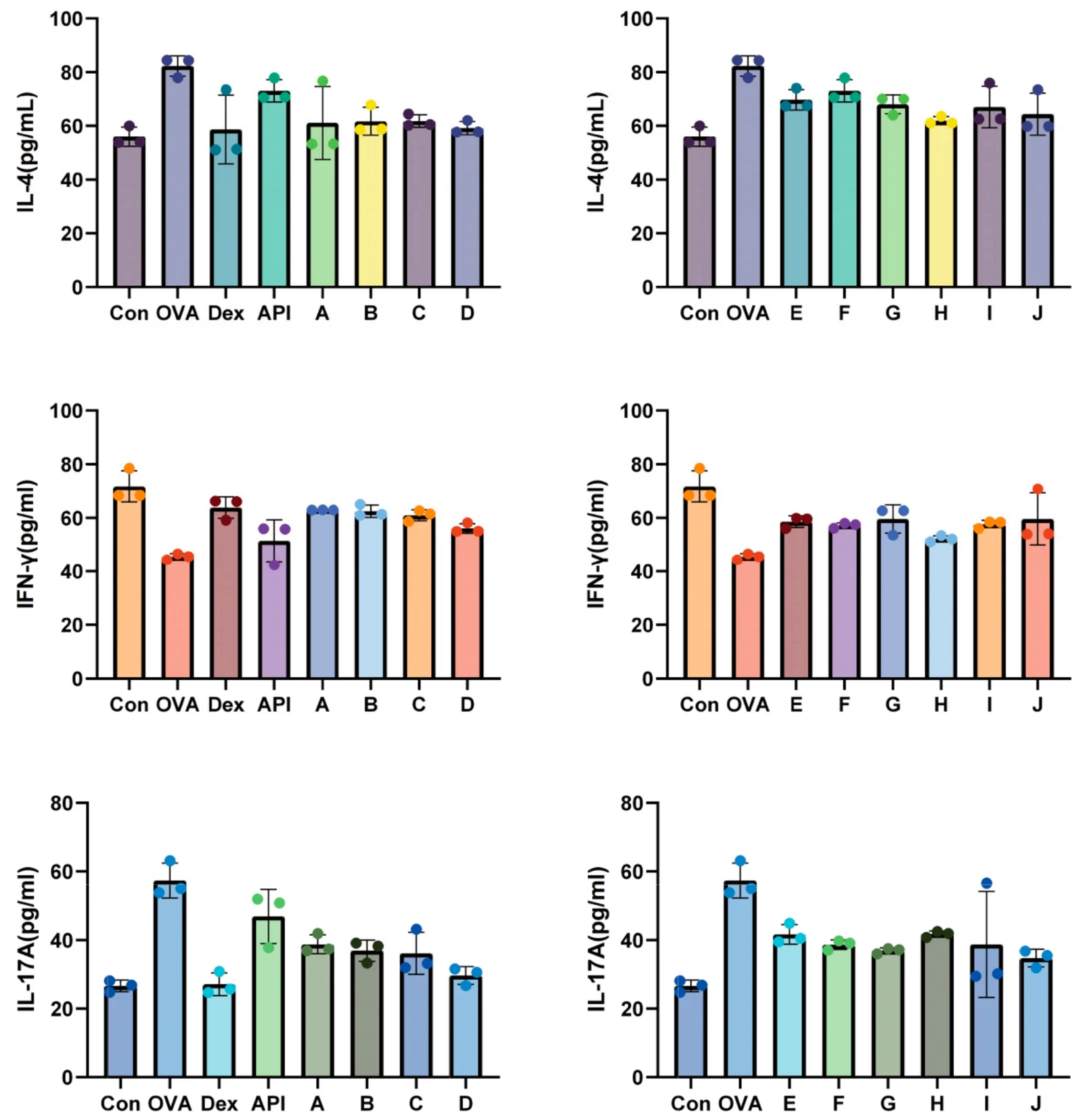
Expression levels of IL-4, IFN-γ, and IL-17A in mouse BALF. Con, normal control; OVA, ovalbumin-induced asthma model; Dex, dexamethasone; API, andrographolide; A–J, synthesized API derivatives. The OVA group presented dramatically increased IL-4 and IL-17A levels and decreased IFN-γ levels compared with the Con group (*p* < 0.01). Dex robustly corrected the Th1/Th2/Th17 immune imbalance (*p* < 0.01), and API also partially improved cytokine dysregulation (*p* < 0.05). All derivatives showed superior regulatory capacity compared with API: triethyl alkyl derivative D achieved IL-4 inhibition comparable to Dex; tri-butyryl acylated derivative J showed IL-17A downregulation activity equivalent to that of Dex; H and I also displayed prominent cytokine modulation, whereas di-substituted derivatives E and G showed relatively weaker efficacy. Tri-substituted derivatives exhibited stronger immunomodulatory activity than their di-substituted counterparts. Data are presented as mean ± SD. Error bars represent the standard deviation, and each dot represents one technical replicate (*n* = 3).

Abnormal activation of the Th2 immune response, accompanied by a Th1/Th17 imbalance, is considered a key immunological feature of OVA-induced allergic asthma. Among these, IL-4, IFN-γ, and IL-17A, representative cytokines associated with Th2, Th1, and Th17 cells, respectively play important roles in the regulation of airway inflammation [21]. As shown in Figure 5, compared with the Con group, IL-4 and IL-17A levels in BALF from OVA-sensitized mice were significantly elevated (*p* < 0.01), while IFN-γ levels were significantly reduced (*p* < 0.01), suggesting that OVA sensitization and challenge successfully induced an imbalance in the expression of inflammation-related cytokines. Compared with the OVA group, the Dex group significantly reduced IL-4 and IL-17A levels and upregulated IFN-γ expression (*p* < 0.01), indicating its potent anti-inflammatory and immunomodulatory effects. The API group also improved the abnormal expression of the aforementioned cytokines to varying degrees (*p* < 0.05), suggesting that API possesses certain anti-inflammatory activity. Among the API derivative intervention groups, most derivatives showed a trend toward greater improvement than the API group. Among them, the alkylated derivative Group D exhibited a relatively pronounced regulatory effect on IL-4, IL-17A, and IFN-γ. Among the acylated derivatives, Groups H and J also demonstrated good improvement effects, while Groups E, I, and G exhibited relatively weaker effects. Furthermore, Group D’s inhibitory effect on IL-4 and Group J’s downregulatory effect on IL-17A were comparable to those of the Dex group. These results suggest that structural modification of API can further enhance its ability to correct OVA-induced imbalances in pro-inflammatory cytokines, with certain alkylated and acetylated derivatives exhibiting significant anti-inflammatory and immunomodulatory activity.

### 2.6. Effects on IgE Levels in the Serum of Asthma Model Mice

**Figure 6:**
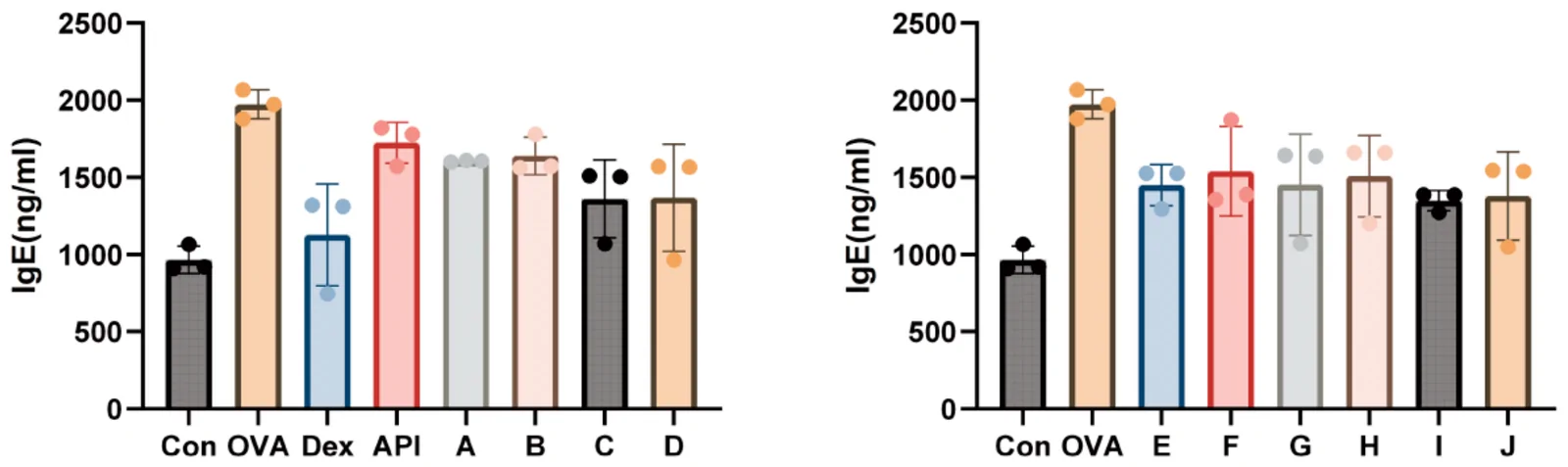
IgE levels in mouse serum. Serum IgE was significantly upregulated in the OVA group versus the Con group (*p* < 0.01). Dex and API both lowered IgE concentration (*p* < 0.01, *p* < 0.05). All apigenin derivatives exerted stronger anti-allergic activity than API (*p* < 0.05). For alkylated derivatives, tri-substituted C, D reduced IgE more obviously than di-substituted A, B (*p* < 0.01). For acylated derivatives, F, J showed the most significant IgE-lowering effect (*p* < 0.01), followed by H, E, G, I. Bearing identical acyl carbon chain lengths, tri-substituted derivatives exhibited better anti-allergic performance than di-substituted ones, though all derivatives were less potent than Dex. Data are presented as mean ± SD. Error bars represent the standard deviation, and each dot represents one technical replicate (*n* = 3).

IgE is a key effector molecule in the Th2-type immune response; changes in its levels reflect the severity of the body’s allergic reaction. Therefore, total serum IgE levels are often used as an important indicator for evaluating OVA-induced allergic asthma models and the antiallergic effects of test drugs [22,23]. As shown in Figure 6, compared with the Con group, serum total IgE levels were significantly elevated in mice in the OVA group (*p* < 0.01), suggesting that OVA sensitization and challenge successfully induced an allergic response, further validating the successful establishment of the allergic asthma model. Compared with the OVA group, the Dex group significantly reduced serum total IgE levels (*p* < 0.01), and the API group also reduced IgE expression to varying degrees (*p* < 0.05), indicating that both possess certain antiallergic effects. Among the API derivative intervention groups, most derivatives exhibited a more pronounced IgE-lowering effect than the API group; however, the overall improvement was still weaker than that observed in the Dex group. Derivatives with different structural modifications showed certain differences in reducing serum IgE levels. Among them, the alkylated derivatives in Groups C and D exhibited a more pronounced effect on improving serum IgE levels (*p* < 0.01), while Groups A and B also significantly reduced IgE levels (*p* < 0.05). Overall, tri-substituted alkylated derivatives showed a better trend of improvement. Among the acylated derivatives, Groups F and J exhibited a particularly pronounced downregulating effect on IgE levels (*p* < 0.01), while Groups H, E, G, and I also reduced serum IgE levels in model mice to varying degrees (*p* < 0.05). Under conditions of identical acyl carbon chain lengths, the tri-substituted derivatives generally showed a more favorable trend in improvement. These results suggest that structural modification of API can further enhance its ability to alleviate OVA-induced abnormal elevations in serum IgE levels, with some alkylated and acetylated derivatives exhibiting good antiallergic activity.

### 2.7. Effects on the Expression Levels of ROS, MDA, and SOD in Lung Tissue from Asthma Model Mice

**Figure 7:**
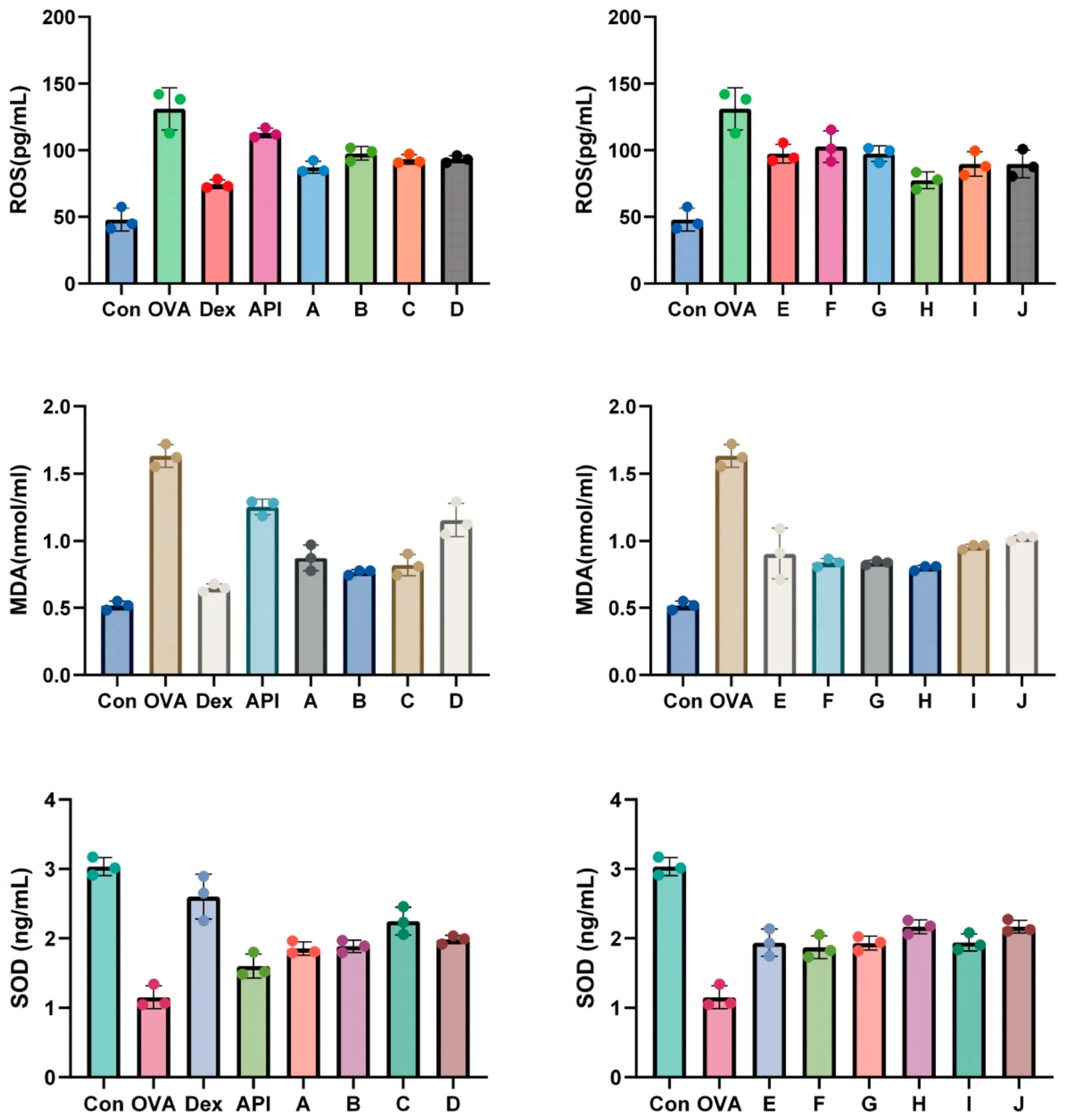
Levels of ROS, MDA, and SOD in mouse lung lavage fluid. OVA exposure induced obvious oxidative stress injury: ROS and MDA contents rose sharply, while SOD activity declined remarkably relative to the Con group (*p* < 0.01). Dex exerted the strongest antioxidant effect (*p* < 0.01), and API alleviated oxidative damage moderately. All derivatives showed better antioxidant capacity than raw API. Alkylated tri-substituted B, C, D prominently enhanced SOD activity and eliminated excess ROS (*p* < 0.01); tri-acylated H, I, J displayed outstanding MDA scavenging ability, while di-substituted E, F, G produced mild antioxidant improvement. Tri-substituted derivatives had stronger antioxidant activity than di-substituted analogs. Data are presented as mean ± SD. Error bars represent the standard deviation, and each dot represents one technical replicate (*n* = 3).

Oxidative stress plays a significant role in the onset and progression of OVA-induced allergic asthma, with ROS, MDA, and SOD serving as key indicators of oxidative stress levels, the degree of lipid peroxidation, and antioxidant capacity, respectively [24,25]. As shown in Figure 7, compared with the Con group, ROS and MDA levels were significantly elevated in the lung tissue of mice in the OVA group, while SOD activity was significantly reduced (*p* < 0.01), suggesting that OVA induces significant oxidative stress damage in mouse lung tissue. Compared with the OVA group, the Dex group significantly reduced ROS and MDA levels and increased SOD activity (*p* < 0.01). The API group also improved the aforementioned oxidative stress markers to varying degrees, but the effect was relatively weaker. Among the apigenin derivative intervention groups, most derivatives exhibited antioxidant effects superior to those of the API group, but the overall improvement was still weaker than that of the Dex group. Derivatives with different structural modifications exhibited certain differences in regulating oxidative stress markers. Among them, the alkylated derivatives in Groups B, C, and D showed a more pronounced effect in increasing SOD activity and reducing ROS levels, while Group A exhibited a relatively weaker effect; the acylated derivatives in Groups H, I, and J were particularly effective in reducing MDA levels, and Groups E, F, and G also improved oxidative stress markers to varying degrees. These results suggest that structural modification of apigenin can further enhance its protective effects against OVA-induced oxidative stress damage, with certain alkylated and acetylated derivatives exhibiting particularly strong antioxidant activity.

### 2.8. Effects on Pathological Changes in Lung Tissue of Asthma Model Mice

**Figure 8:**
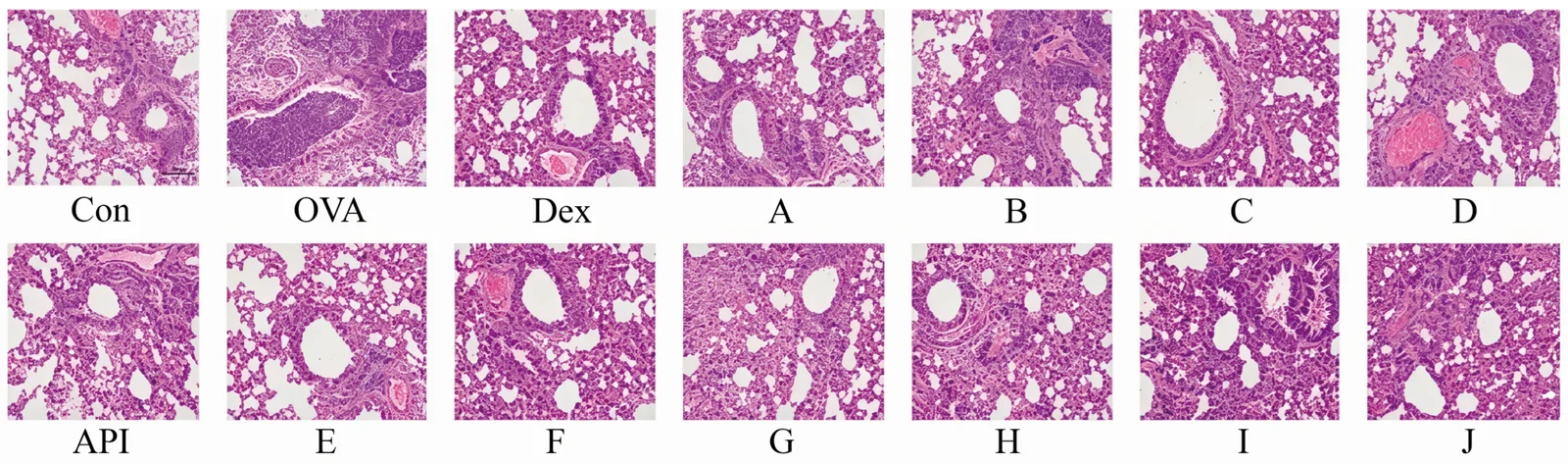
HE-stained pathological sections of mouse lung tissue. The Con group showed intact airway epithelium, normal alveolar structure and rare inflammatory infiltration. The OVA group exhibited severe mucosal edema, massive inflammatory cell infiltration, airway stenosis and alveolar disorganization. Dex nearly restored lung tissue morphology to a normal level. API relieved pathological lesions moderately, yet mild inflammation still existed. All derivatives ameliorated OVA-triggered lung injury more effectively than API. Tri-substituted D (triethyl alkyl) and H (triacetyl) achieved the most prominent remission of epithelial damage and inflammatory cell infiltration, followed by B, F, I, J; di-substituted compounds A, C, E, G produced weaker pathological improvement.

HE staining results were used to evaluate pathological changes in lung tissue from mice with OVA-induced allergic asthma and the effects of API and its derivatives [26]. As shown in Figure 8, the lung tissue of mice in the Con group exhibited intact structure, continuous airway epithelium, no obvious mucosal edema, no significant inflammatory cell infiltration around the airways or blood vessels, and clear alveolar structure. Mice in the OVA group exhibited typical asthma-like pathological changes, including disruption of airway epithelial structure, mucosal edema, massive inflammatory cell infiltration around the airways and blood vessels, narrowing or obstruction of the airway lumen, as well as disorganized alveolar structure and thickened airway walls. Compared with the OVA group, the Dex group showed significant improvement in the aforementioned pathological changes, as evidenced by relatively intact airway epithelial structure, reduced mucosal edema, decreased inflammatory cell infiltration, restored airway patency, and lung tissue structure approaching normal. The API group also alleviated lung tissue damage to some extent, but mild inflammatory cell infiltration and mucosal edema were still observable. Following intervention with API derivatives, all treatment groups showed varying degrees of improvement in OVA-induced pulmonary pathological damage. The overall improvement was superior to that of the API group but inferior to that of the Dex group. Among them, Groups B, D, and H demonstrated a more pronounced improvement in airway structural damage and inflammatory cell infiltration, while the remaining derivative groups also alleviated airway mucosal edema and inflammatory cell infiltration to varying degrees and improved alveolar structural disorganization.

API has attracted considerable attention because of its anti-inflammatory and antioxidant activities. However, its clinical application is limited by poor water solubility, low bioavailability, and rapid metabolism [27,28]. Structural modification has therefore become an effective strategy for improving its pharmacological properties, with previous studies mainly focusing on substitutions at the A, B, and C rings of the flavonoid skeleton [29,30]. Various alkyl, acyl, methoxy, and halogen substituents have been reported to enhance the biological activity and bioavailability of flavonoids [31,32,33]. Since allergic asthma is closely associated with Th1/Th2/Th17 immune imbalance, excessive production of IL-4, IL-17A, and IgE, together with insufficient IFN-γ expression, contributes to airway inflammation and disease progression [34,35,36]. Based on these findings, API was selected as the lead compound, and 10 di- and tri-substituted derivatives were synthesized by selectively modifying the 5-, 7-, and 4′-hydroxyl groups, including six novel compounds (B, D, F, H, I, and J), whereas compounds A [37], C [38], E [39], and G [40] had been previously reported. The derivatives were synthesized under mild conditions and structurally characterized by FT-IR, ^1^H-NMR and ^13^C-NMR spectroscopy. O-Alkyl and O-acyl substituents were introduced to optimize steric, electronic, and lipophilic properties while preserving the flavonoid scaffold. Although these modifications may reduce intrinsic aqueous solubility, all compounds were readily formulated in 1% DMSO aqueous solution without visible precipitation, suggesting that differences in biological activity were unlikely to result from solubility under the experimental conditions.

In the OVA-induced murine asthma model, API and its derivatives exhibited no apparent toxicity at 20 mg/kg and significantly ameliorated OVA-induced body weight loss, increased lung and spleen indices, inflammatory responses, oxidative stress, and pathological lung injury. Compared with native API, the derivatives more effectively reduced IL-4, IL-17A, serum IgE, ROS, and MDA levels while increasing IFN-γ expression and SOD activity, indicating improved regulation of immune responses and oxidative stress. Histopathological analysis further demonstrated marked attenuation of airway epithelial injury, inflammatory cell infiltration, and alveolar structural disruption. Notably, the tri-substituted and long-chain derivatives displayed superior overall efficacy, with compounds D and F producing therapeutic effects comparable to those of dexamethasone. These findings suggest that rational structural modification substantially enhances the anti-inflammatory and antioxidant activities of API and improves its therapeutic efficacy against allergic asthma, possibly through regulation of inflammatory cytokine networks, restoration of immune homeostasis, and attenuation of oxidative stress. Nevertheless, the physicochemical characteristics, pharmacokinetic behavior, detailed molecular mechanisms, and systematic structure-activity relationships of these derivatives remain to be elucidated and warrant further investigation to support their future therapeutic development.

## 3. Materials and Methods

### 3.1. Materials and Reagents

All raw materials and reagents used in the experiments were of analytical grade and used without further purification. Chemical reagents, including apigenin (purity ≥ 98%), acetyl chloride, butyryl chloride, propionyl chloride, diethyl sulfate, dimethyl sulfate, acetone, ethyl acetate, petroleum ether, dimethyl sulfoxide (DMSO), anhydrous potassium carbonate, etc., were of analytical grade and purchased from Shanghai Aladdin Biochemical Technology Co., Ltd. (Shanghai, China). Ovalbumin was obtained from Shanghai Aladdin Biochemical Technology Co., Ltd. (Shanghai, China), and dexamethasone was purchased from Guangdong Nanguo Pharmaceutical Co., Ltd. (Guangzhou, China). All assay kits applied in this study were supplied by Jiangsu Meimian Industrial Co., Ltd. (Yancheng, China), including detection kits for ROS, MDA and SOD, as well as mouse IL-4, IL-17A, IFN-γ and IgE ELISA kits. SPF-grade female BALB/c mice aged 6–8 weeks were used as experimental animals and purchased from Liaoning Changsheng Biotechnology Co., Ltd. (Shenyang, China; Production License No.: SCXK (Liao) 2020-0001).

### 3.2. Synthesis of Alkylated Derivatives of Apigenin

**Figure 9:**
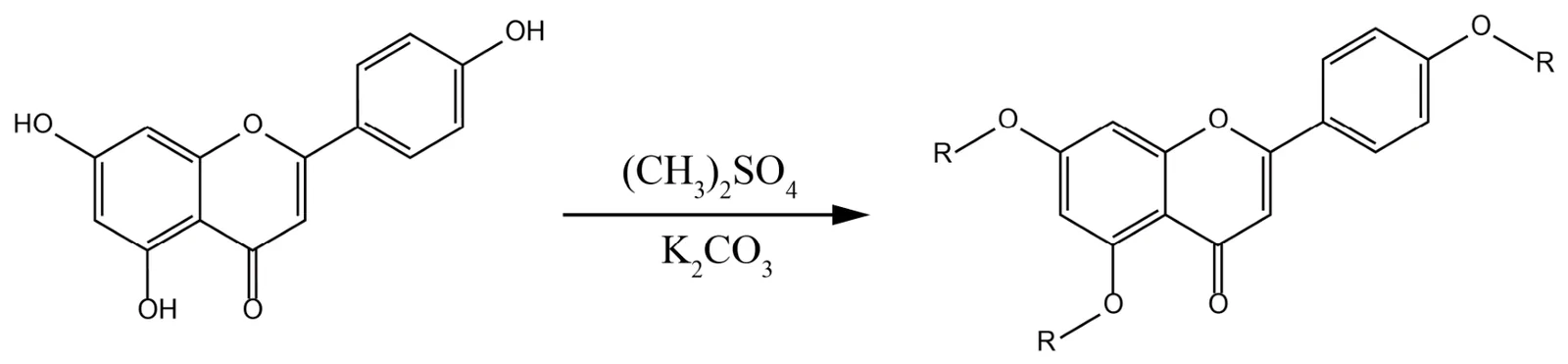
Synthesis route for alkylated apigenin derivatives.

Reaction Mechanism: Under basic conditions, K_2_CO_3_ promotes the deprotonation of the 4′-OH and 7-OH groups in API, enhancing its nucleophilicity. The phenoxy anion of dimethyl sulfate attacks the partially positively charged methyl carbon in the dialkyl sulfate as a nucleophile, forming a transition state. In the leaving group elimination transition state, the ROSO_2_O^−^ anion departs as the leaving group, ultimately forming a methyl ether bond (-O-CH_3_) while simultaneously generating sulfate byproducts (K_2_SO_4_). See Figure 9.

#### 3.2.1. Synthesis of Apigenin via a Di-Substituted -O-Alkylation Reaction

In the experiment, API was used as the starting material, and alkylation reactions were carried out using dimethyl sulfate and diethyl sulfate as alkylating agents, respectively. During the reaction, 100 mL of anhydrous acetone solution was added to a round-bottom flask, followed by the API (5 mmol, 1.35 g), and the mixture was stirred thoroughly for 10 min. Anhydrous potassium carbonate (30 mmol, 4.14 g) was added, and the mixture was stirred at room temperature for 30 min. Subsequently, dimethyl sulfate/diethyl sulfate (20 mmol) was slowly added dropwise to the round-bottom flask, and the reaction was allowed to proceed at room temperature for 6 h, with continuous monitoring via TLC until the reaction was complete. After the reaction was complete, the mixture was cooled to room temperature, and the acetone was evaporated under reduced pressure. Water was added to the residue and stirred thoroughly, followed by filtration under vacuum to yield a pale yellow crude product. The crude product was dissolved in anhydrous ethanol, hot-filtered, allowed to stand and cool, and recrystallized twice. After filtration, the product was dried under vacuum at 60 °C to constant weight to obtain derivatives A and B.

#### 3.2.2. Synthesis of Apigenin via a Tri-Substituted -O-Alkylation Reaction

In the experiment, API was used as the starting material, and alkylation reactions were carried out using dimethyl sulfate and diethyl sulfate as alkylating agents, respectively. During the reaction, 100 mL of anhydrous acetone solution was added to a round-bottom flask, followed by the API (5 mmol, 1.35 g), and the mixture was stirred thoroughly for 10 min. Anhydrous potassium carbonate (45 mmol, 6.22 g) was then added, and the mixture was stirred at room temperature for 30 min. Next, dimethyl sulfate/diethyl sulfate (30 mmol) was slowly added dropwise to the round-bottom flask, and the reaction was allowed to proceed at room temperature for 3 h. Subsequently, 0.95 mL of dimethyl sulfate (10 mmol) and an appropriate amount of 10% NaOH solution were added to adjust the alkalinity, and the reaction was continued for 2 h, monitoring the reaction continuously via TLC until complete. Upon completion of the reaction, the mixture was cooled to room temperature, and acetone was removed by reduced-pressure evaporation. Deionized water was added to the resulting residue, followed by sufficient stirring and vacuum filtration to afford a pale yellow crude product. The crude product was dissolved in anhydrous ethanol, subjected to hot filtration, then allowed to stand and cool naturally. After two rounds of recrystallization, the product was collected by vacuum filtration and dried under vacuum at 60 °C until a constant weight was achieved, affording derivatives C and D.

### 3.3. Synthesis of Acylated Apigenin Derivatives

**Figure 10:**
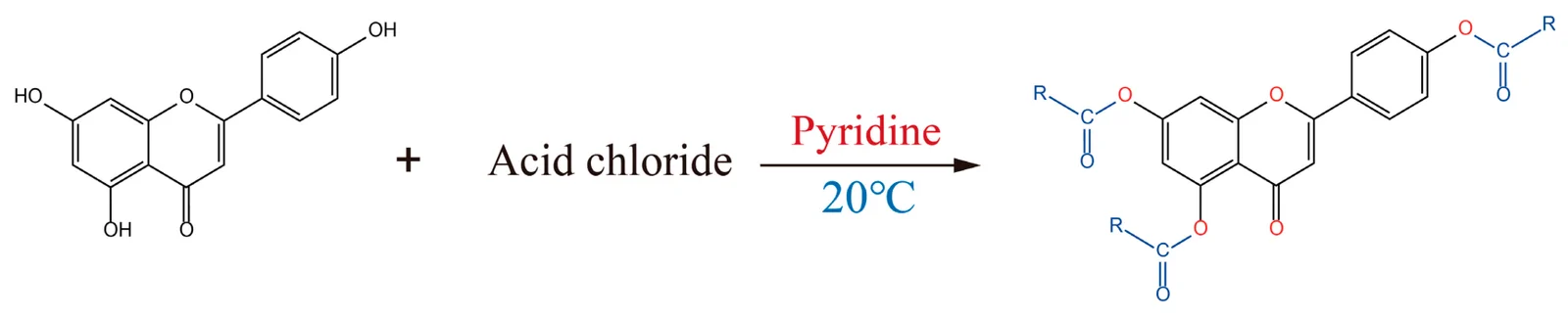
Synthesis Routes for Acetylated Derivatives of Apigenin.

Reaction Mechanism: The phenolic hydroxyl group (-OH) in deprotonated API exhibits acidic properties; pyridine abstracts a proton from the phenolic hydroxyl group, forming a phenoxy anion (nucleophile). Nucleophilic Attack on the Acyl Chloride: The phenoxy anion, acting as a nucleophile, attacks the carbonyl carbon (electrophilic center) of the acyl chloride (R-COCl), forming a tetrahedral intermediate. Departing group elimination: The tetrahedral intermediate undergoes rearrangement, and the chloride anion (Cl^−^) departs as the leaving group, ultimately forming an ester bond (-O-CO-R). At the same time, pyridine donates a proton to form a pyridine salt (byproduct). See Figure 10.

#### 3.3.1. Synthesis of Apigenin via a Di-Substituted -O-Acylation Reaction

Accurately weigh apigenin (5 mmol, 1.35 g) and dissolve it in 100 mL of anhydrous acetone; add pyridine (30 mmol), and cool the mixture to 0 °C in an ice bath. Acetyl chloride, propionyl chloride, and butyryl chloride (15 mmol) were then slowly added dropwise. After completion of the titration, the ice bath was removed, and the mixture was stirred continuously under an electromagnetic stirrer at room temperature for 6 h, with the reaction monitored by TLC. Upon completion of the reaction, the mixture was quenched with ice-water, the organic layer was extracted with ethyl acetate, the organic phases were combined, and the crude product was obtained by concentration under reduced pressure. The crude product was stirred in hot ethanol at 60 °C until completely dissolved; distilled water was slowly added dropwise until the solution became slightly turbid, after which it was reheated until clear. The mixture was allowed to stand at room temperature and then refrigerated overnight at 4 °C to promote crystallization. The solid was isolated by vacuum filtration, washed with cold ethanol, and dried to yield derivatives E, F, and G.

#### 3.3.2. Synthesis of Apigenin via a Tri-Substituted -O-Acylation Reaction

Accurately weigh apigenin (5 mmol, 1.35 g) and dissolve it in 100 mL of anhydrous acetone; add pyridine (45 mmol), and cool the mixture to 0 °C in an ice bath. Acetyl chloride, propionyl chloride, and butyryl chloride (22.5 mmol) were then slowly added dropwise. After completion of the titration, the ice bath was removed, and the mixture was stirred continuously under magnetic stirring at room temperature for 12 h, with the progress monitored by TLC. After the reaction was complete, the mixture was quenched with ice-water, the organic layer was extracted with ethyl acetate, the organic phases were combined, and the mixture was concentrated under reduced pressure to yield the crude product. The crude product was placed in hot ethanol at 60 °C and stirred until completely dissolved; distilled water was slowly added dropwise until the solution became slightly turbid, after which it was reheated until clear. The mixture was allowed to stand at room temperature and then refrigerated overnight at 4 °C to allow crystallization. The solid was isolated by vacuum filtration, washed with cold ethanol, and dried to yield derivatives H, I, and J.

### 3.4. Structural Characterization of Derivatives A–J

#### 3.4.1. Fourier-Transform Infrared Spectroscopy

Weigh appropriate amounts of the solid forms of 10 API derivatives and an appropriate amount of KBr powder, place them together in an agate mortar, grind them into a very fine powder, and compress them into tablets. Infrared spectroscopic analysis was performed using a Fourier transform infrared spectrometer (Varian 640-IR, Varian Medical Systems, Palo Alto, California, USA). This was used to detect characteristic absorption peaks of the compounds’ functional groups and to verify structural changes through shifts in peak positions.

#### 3.4.2. Nuclear Magnetic Resonance Spectroscopic Analysis

Nuclear magnetic resonance (NMR) spectroscopy is an important technique for structural characterization of organic compounds. Approximately 1 mg of the dried API derivative was dissolved in deuterated dimethyl sulfoxide (DMSO-d6) and transferred into an NMR tube. The ^1^H-NMR and ^13^C-NMR spectra were recorded using an NMR spectrometer (Agilent 400 NMR, Agilent Technologies, Inc., Santa Clara, California, USA). The chemical structures of the synthesized derivatives were confirmed by analyzing the chemical shifts, coupling constants, signal multiplicities, and integral values in the ^1^H-NMR spectra together with the chemical shifts and signal assignments in the ^13^C-NMR spectra, thereby providing information on the hydrogen and carbon environments, functional groups, and molecular framework of the compounds.

### 3.5. Study on the Oxidative Stress Activity of Derivatives A–J

#### 3.5.1. Animal Grouping and Dosage Administration Methods

Following structural characterization of compounds A–J, animal experiments related to oxidative stress were conducted. A total of 112 female SPF-grade BALB/c mice were used in the experiment. Following the principle of randomization, the mice were numbered according to body weight and divided into 14 groups of 8 mice each using a randomization table. The groups were as follows: control group (Con), model group (OVA), dexamethasone-positive control group (Dex), apigenin group (API), and compound A–J groups. The experimental process consisted of sensitization, challenge, and treatment phases. After a 7 day acclimatization period, a bronchial asthma model was established. On days 1 and 7 of the modeling process, intraperitoneal injections were administered: the control group received 200 μL of saline, while the remaining groups received 200 μL of a gel solution containing 50 μg of OVA and 1 mg of aluminum hydroxide for sensitization. On days 14, 15, 20, and 21, the control group received 40 μL of saline via instillation; mice in the asthma group, positive control group, API group, and compound A–J groups received 40 μL of a saline solution containing 50 μg of OVA via intranasal instillation for challenge. During treatment, from day 14 to day 21, the positive control group received 1 mg/kg orally once daily for 7 consecutive days. The control group and asthma group received intraperitoneal injections of 200 μL of a 1% DMSO solution, while the apigenin group and the compound A–J groups received intraperitoneal injections of 200 μL of a solution containing 20 mg/kg of apigenin or compounds A–J dissolved in 1% DMSO.

#### 3.5.2. Collection of Mouse Serum, Lung Tissue, and Bronchoalveolar Lavage Fluid

Mouse body weights were measured on days 1, 4, 8, 12, 16, 20, and 23 following OVA sensitization. On day 23, the mice were euthanized, and the weights of their lungs and spleens were recorded. Prior to euthanasia, the mice were anesthetized with sodium pentobarbital; after they lost consciousness, blood was collected via the orbital venous plexus and placed in centrifuge tubes [41]. The collected blood was centrifuged at 4 °C and 4000 r/min for 5 min, and the supernatant serum was then transferred to a new centrifuge tube, labeled, and set aside. After serum collection, the skin of each mouse’s chest was incised to fully expose the thoracic cavity and lung tissue. Using the bronchopulmonary lavage method, a small incision was made in the mid-trachea, and a sterile catheter was inserted. A 1 mL syringe was used to draw up 0.9% sterile saline pre-chilled to 4 °C, and the lungs were subjected to bronchoalveolar lavage [42]. The lavage solution was injected in three separate doses of 0.3 mL, 0.3 mL, and 0.4 mL, respectively, to ensure thorough perfusion of the lung tissue while avoiding damage caused by excessive volume or too rapid a flow rate. After the final injection, the specimen was allowed to stand for 30 s, and the lavage solution was slowly aspirated back, ensuring that at least 80% of the volume was recovered. The collected lavage fluid was placed in a centrifuge tube and centrifuged for 5 min at 4 °C and 3000 r/min. After centrifugation, the supernatant was collected and stored at −80 °C for future use. Once the lavage fluid collection was complete, a portion of the lung tissue was placed in a cryopreservation tube and stored at −80 °C, while another portion was fixed in 4% paraformaldehyde for subsequent histopathological analysis.

#### 3.5.3. Measurement of Inflammatory Cytokines in Bronchoalveolar Lavage Fluid and Serum IgE

The enzyme-linked immunosorbent assay (ELISA) is based on the specific binding of antigens to antibodies and combines the immunological recognition reaction with the catalytic amplification effect of enzymes [43]. The ELISA was used to detect the levels of IgE in mouse serum and IL-4, IL-17A, and IFN-γ in bronchoalveolar lavage fluid. The supernatant samples used in the experiment were removed from a −80 °C freezer and allowed to reach room temperature prior to testing; subsequent procedures were conducted in strict accordance with the instructions for the corresponding mouse ELISA kit. First, the kit and related reagents were removed from a 4 °C refrigerator and allowed to equilibrate at room temperature for 20 min. The required number of microplate strips were removed as needed for the experiment, and unused strips were resealed and stored at 4 °C. Add 50 μL of standard solutions at different concentration gradients to the standard wells; to the sample wells, add 10 μL of the sample to be tested, followed by 40 μL of sample diluent; leave the blank wells empty. After adding the samples, seal the plate with a cover film and incubate at 37 °C for 30 min. After incubation, discard the liquid from the wells. Add washing solution to each well, let stand for 1 min, then vortex to remove excess liquid and blot dry on absorbent paper. Repeat this washing procedure 5 times. Except for the blank wells, add 50 μL of horseradish peroxidase-labeled detection antibody to each well. Seal the plate and continue incubation at 37 °C for 30 min, then wash the plate 5 more times using the method described above. Add 50 μL of color development substrate A and 50 μL of color development substrate B to each well, respectively. Incubate at 37 °C in the dark for 15 min, then add 50 μL of stop solution to each well to terminate the reaction. Measure the absorbance of each well at a wavelength of 450 nm using a microplate reader within 15 min. Plot a standard curve using the absorbance values of the standards, and calculate the concentrations of IgE, IL-4, IL-17A, and IFN-γ in the samples based on the standard curve.

#### 3.5.4. Measurement of Oxidative Stress Levels in Lung Tissue

Lung tissues were rinsed with ice-cold phosphate-buffered saline (PBS, 0.01 M, pH 7.4) to remove residual blood and homogenized in cold PBS (1:9, *w*/*v*). The homogenates were subjected to ultrasonic disruption and centrifuged at 5000× *g* for 10 min at 4 °C. The resulting supernatants were collected for biochemical analysis. Superoxide dismutase (SOD) activity, malondialdehyde (MDA) content, and total intracellular reactive oxygen species (ROS), including superoxide anion (O_2_^−^), hydrogen peroxide (H_2_O_2_) and hydroxyl radical (·OH), were determined using commercial assay kits from Jiangsu Meimian Industrial Co., Ltd. (Yancheng, China), strictly following the manufacturer’s instructions. The ROS detection kit adopted the fluorescent probe method to quantify the comprehensive level of multiple endogenous reactive oxygen species accumulated in lung tissue cells, mainly covering superoxide anion, hydrogen peroxide, and hydroxyl radicals, which are the primary ROS mediators driving airway oxidative injury in the OVA-induced asthma model. The absorbance/fluorescence signals were measured with a microplate reader.

#### 3.5.5. HE Staining

After lung tissue was fixed in 4% paraformaldehyde, it was subjected to routine paraffin embedding and hematoxylin-eosin (HE) staining for histopathological examination. After fixation, the lung tissue was dehydrated using a gradient of ethanol and clarified with xylene, then immersed in paraffin at 60 °C and embedded to form paraffin blocks. The paraffin blocks were subsequently sectioned into 5-μm-thick sections. After spreading and baking the sections, they underwent xylene dewaxing followed by rehydration using a gradient of ethanol. The rehydrated sections were stained with hematoxylin, followed by differentiation and rebluing, and then counterstained with eosin. After staining, the sections were dehydrated using a gradient of ethanol, cleared with xylene, and mounted with neutral resin. Finally, pathological morphological changes in the lung tissue were observed under a light microscope.

### 3.6. Data Processing

Data were analyzed using the mean ± standard deviation. Significant differences between data sets were analyzed using GraphPad Prism 11 software; *p* < 0.05 was considered statistically significant.

## 4. Conclusions

In the experiment, a series of apigenin-related derivatives were synthesized, with yields ranging from 68.16% to 75.08%. In vivo studies demonstrated that API and its derivatives can suppress ovalbumin-induced asthma in mice by reducing the levels of IL-4 and IL-17A in alveolar lavage fluid, increasing IFN-γ expression, lowering ROS and MDA levels in lung tissue, and enhancing SOD activity. Among these, the two derivatives in groups D and F both demonstrated good efficacy in alleviating asthma, with effects comparable to those of dexamethasone, while the activity of the other groups was slightly weaker but still higher than that of the parent compound, apigenin. Substitutions of the same type exhibited certain structure-activity relationships: tri-substituted apioxanthin derivatives were more active than di-substituted ones, and those with long carbon chains were more active than those with short chains. We speculate that this phenomenon can be attributed to the tri-substituted structure, which enables full modification of all three phenolic hydroxyl groups. This modification improves lipophilicity, cell membrane permeability and molecular stability, and strengthens interactions with target sites, ultimately resulting in superior pharmacological effects compared with di-substituted API derivatives. Accordingly, further research on physicochemical properties, including lipophilicity and bioavailability, can be carried out in subsequent studies, followed by experimental verification. Future studies should focus on further elucidating the molecular mechanisms underlying the biological activities of these derivatives. In addition, comprehensive evaluation of their physicochemical properties, pharmacokinetic behavior, metabolic stability, toxicity, and long-term therapeutic efficacy is required. Further structure-activity relationship studies and optimization of the substituent groups may facilitate the development of more potent and safer apigenin-derived lead compounds for the treatment of allergic asthma.

## Data Availability

The original contributions presented in this study are included in the article and Supplementary Materials. Further inquiries can be directed to the corresponding authors.

## Supplementary Materials

The following supporting information can be downloaded at: https://www.mdpi.com/article/10.3390/molecules31162768/s1, In the supplementary files: Pages 1–2: FT-IR, ^1^H-NMR and ^13^C-NMR spectra of apigenin; Pages 3–7: FT-IR spectra of Compounds A–J; Pages 8–12: ^1^H-NMR spectra of Compounds A–J; Pages 13–17: ^13^C-NMR spectra of Compounds A–J.
