# Supplementary material for: Apigenin Derivatives Alleviate OVA-Induced Oxidative Stress in Bronchial Asthma: A Structure-Activity Relationship Study
Source: Molecules. 2026 Aug 9;31(16):2768. doi: 10.3390/molecules31162768 (PMC13515581; doi:10.3390/molecules31162768)

Apigenin Derivatives Alleviate OVA-Induced Oxidative Stress in Bronchial Asthma: A Structure-Activity Relationship Study

Contents

FT-IR、^{1}H-NMR and ^{13}C-NMR spectra of apigenin •••••••••••••• 1-2

FT-IR Spectra of Compounds A-J •••••••••••••••••••••••••••••3-7

^{1}H-NMR Spectra of Compounds A-J ••••••••••••••••••••••••8-12

^{13}C-NMR Spectra of Compounds A-J •••••••••••••••••••••• 13-17

FT-IR spectrum of apigenin

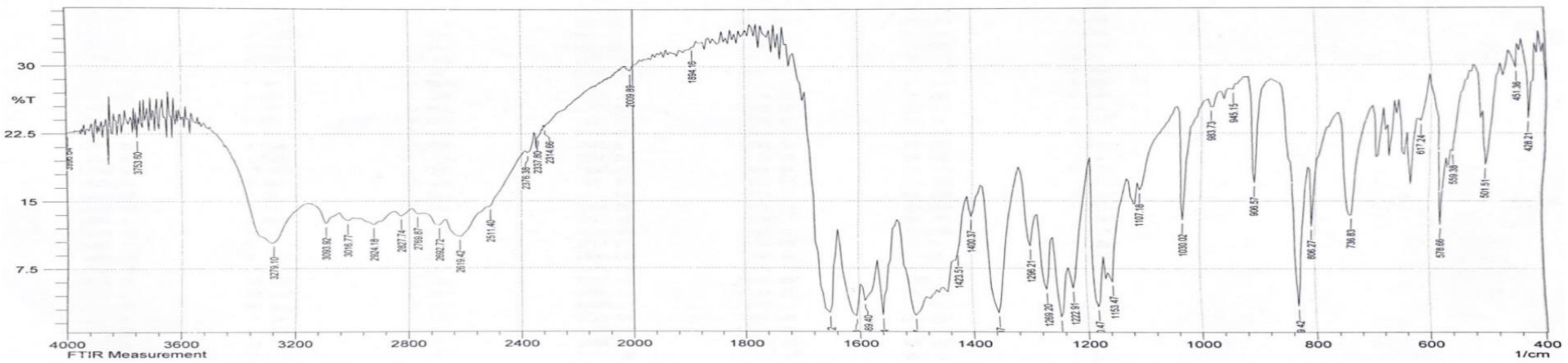

^{1}H-NMR spectrum of apigenin

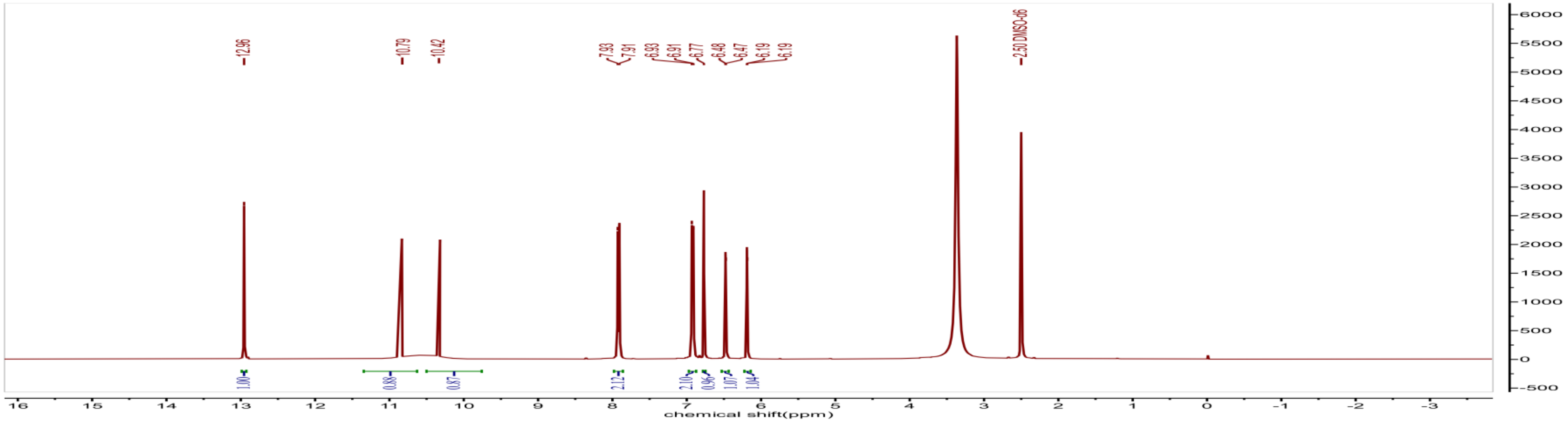

^{13}C-NMR spectrum of apigenin

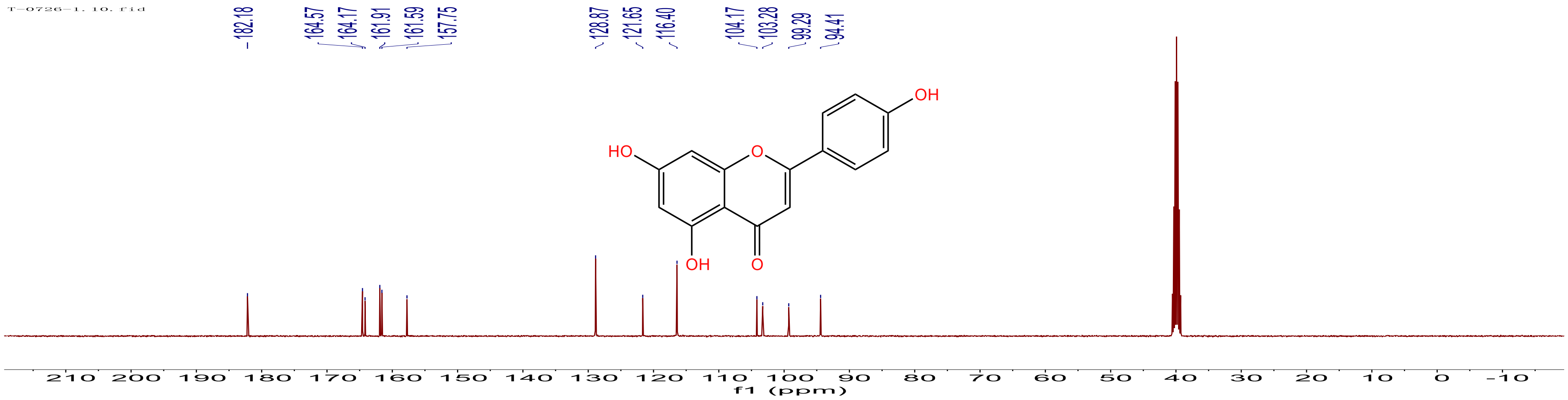

A：7,4’-O-Dimethyl-apigenin

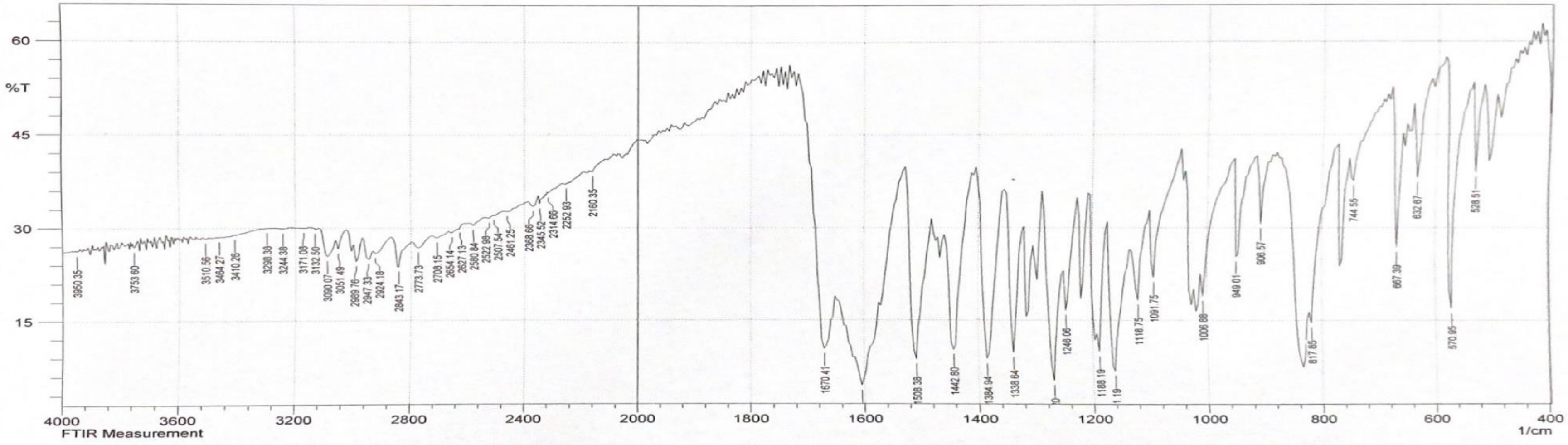

B：7,4’-O-Diethyl-apigenin

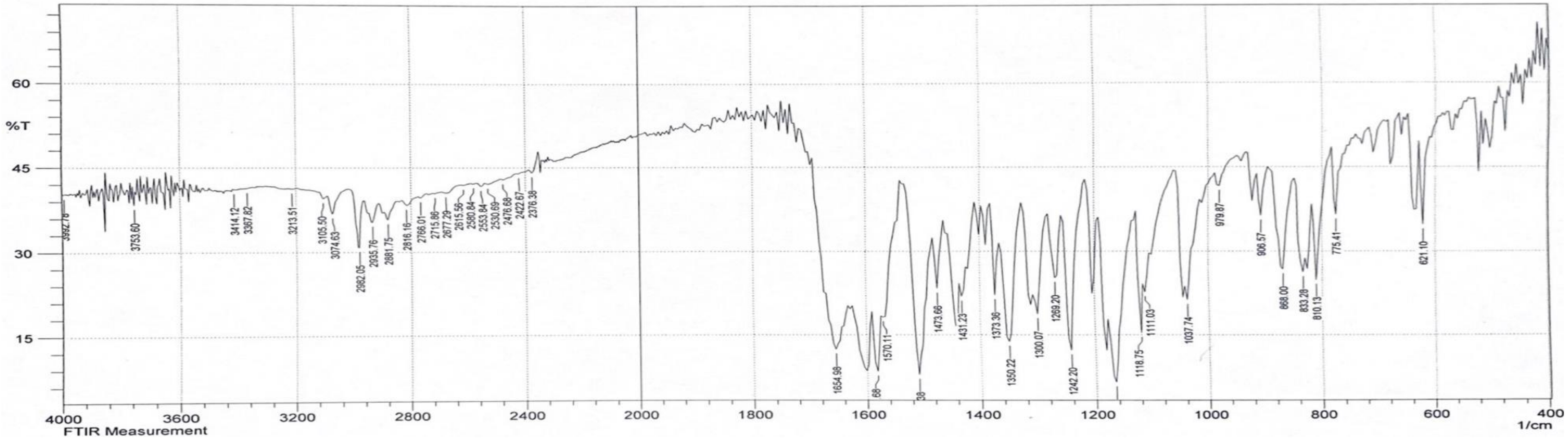

C：5,7,4’-O-Trimethyl-apigenin

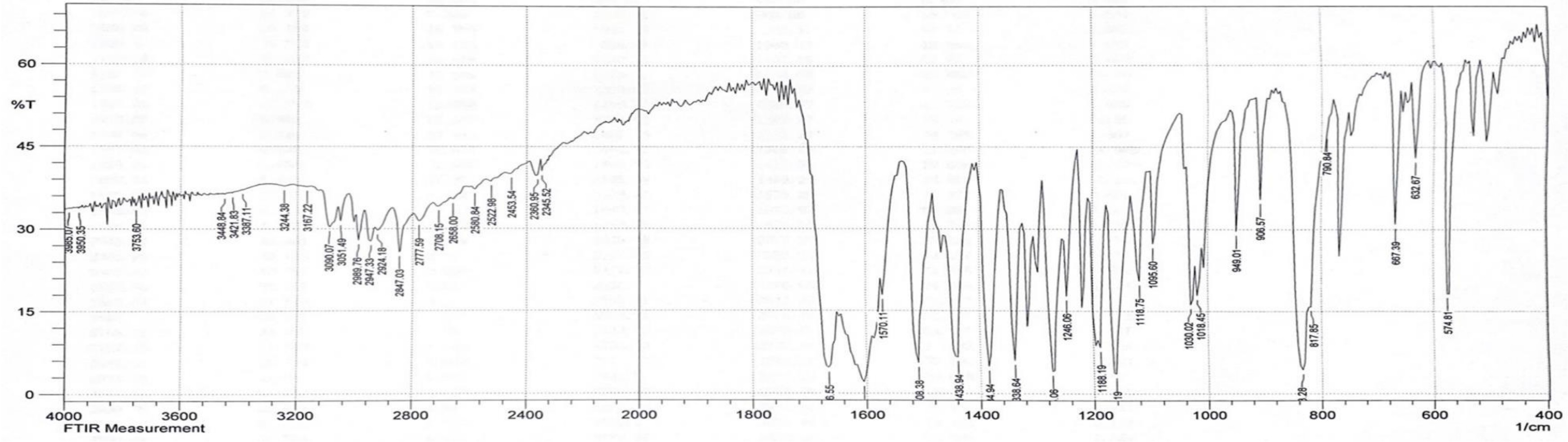

D：5,7,4’-O-Triethyl-apigenin

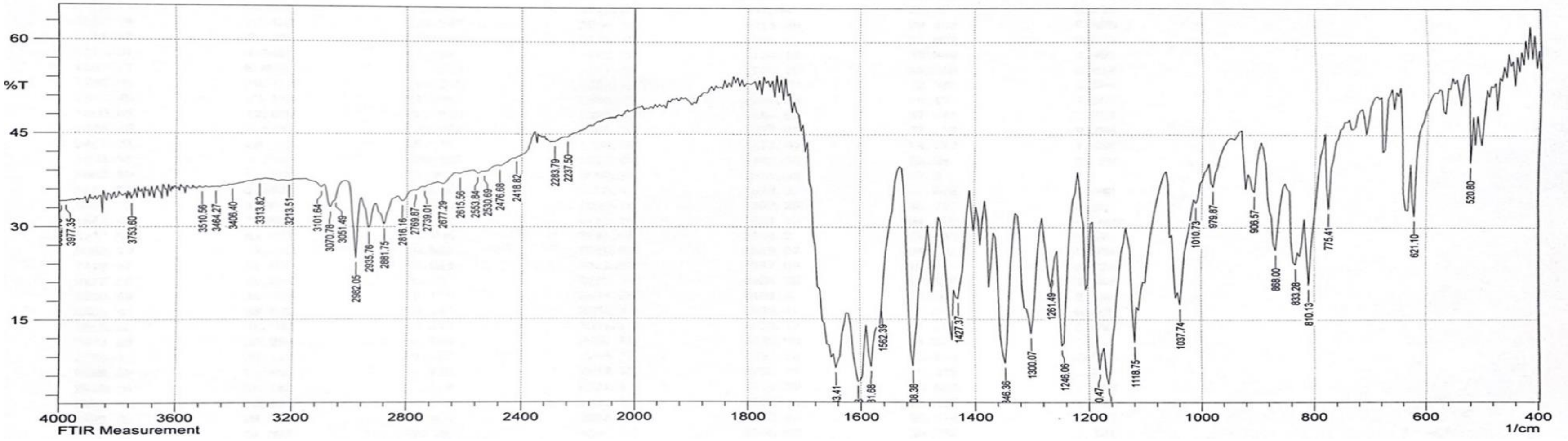

E：7,4’-O-Diacetyl-apigenin

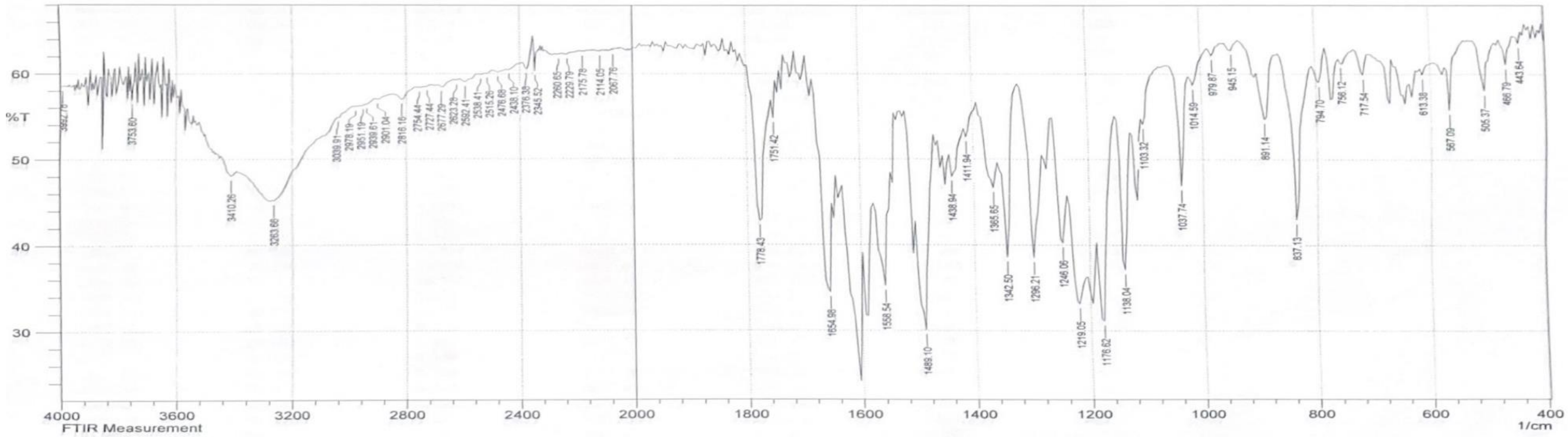

F：7,4’-O-Dipropionyl-apigenin

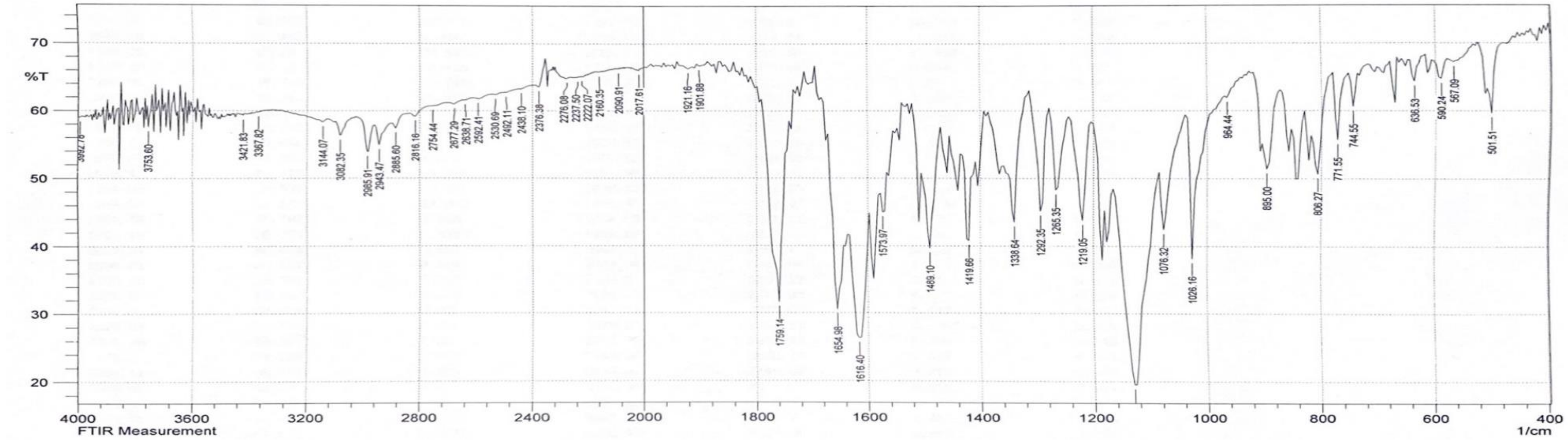

G：7,4’-O-Dibutyryl-apigenin

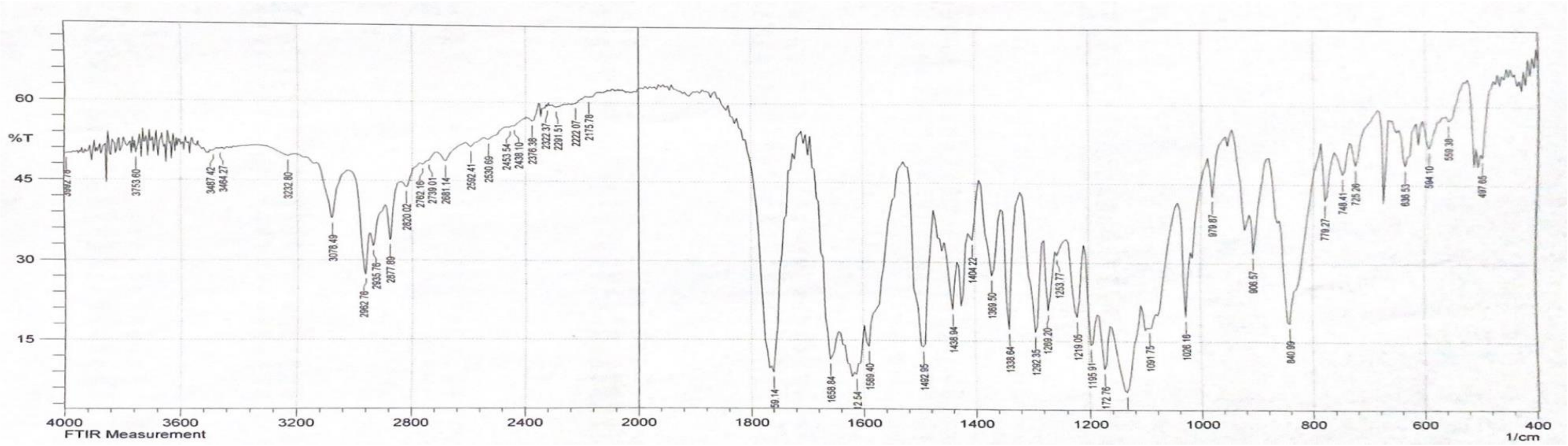

H：5,7,4’-O-Triacetyl-apigenin

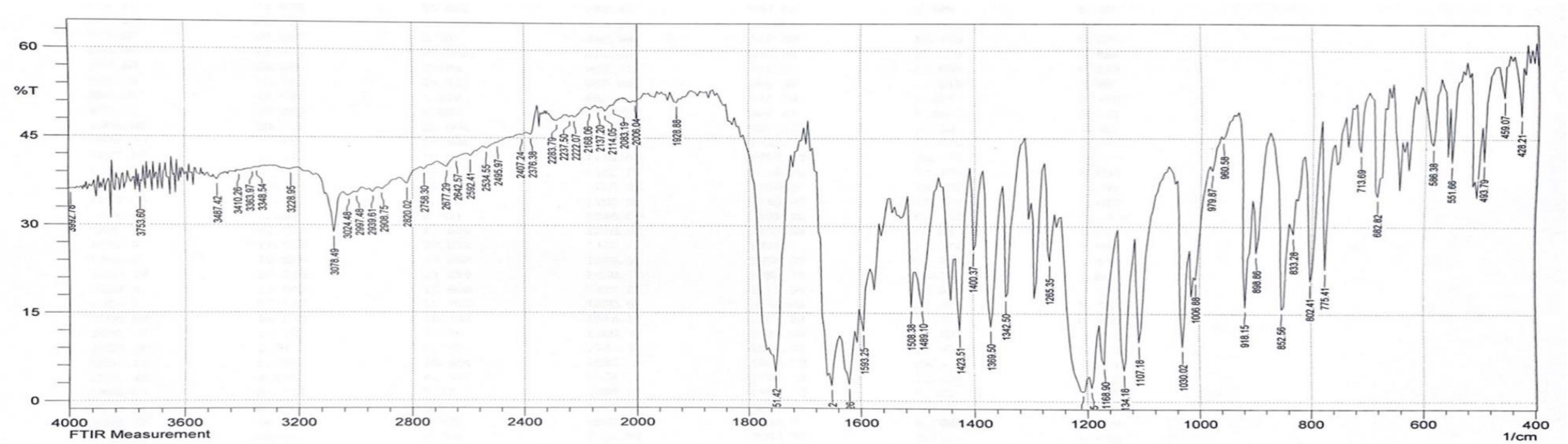

I：5,7,4’-O-Tripropionyl-apigenin

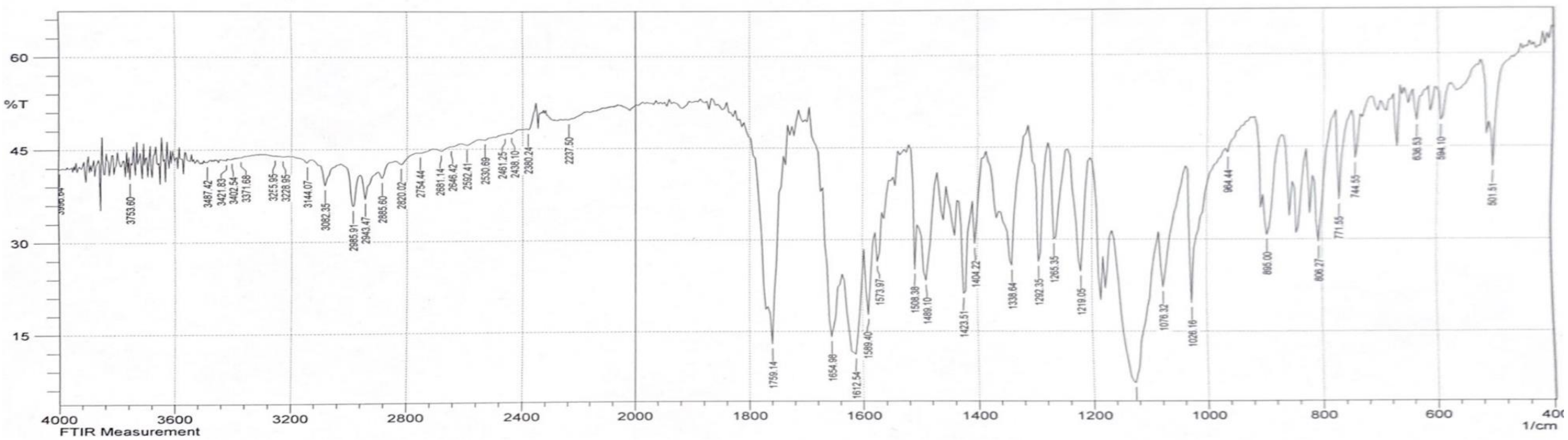

J：5,7,4’-O-Tributyryl-apigenin

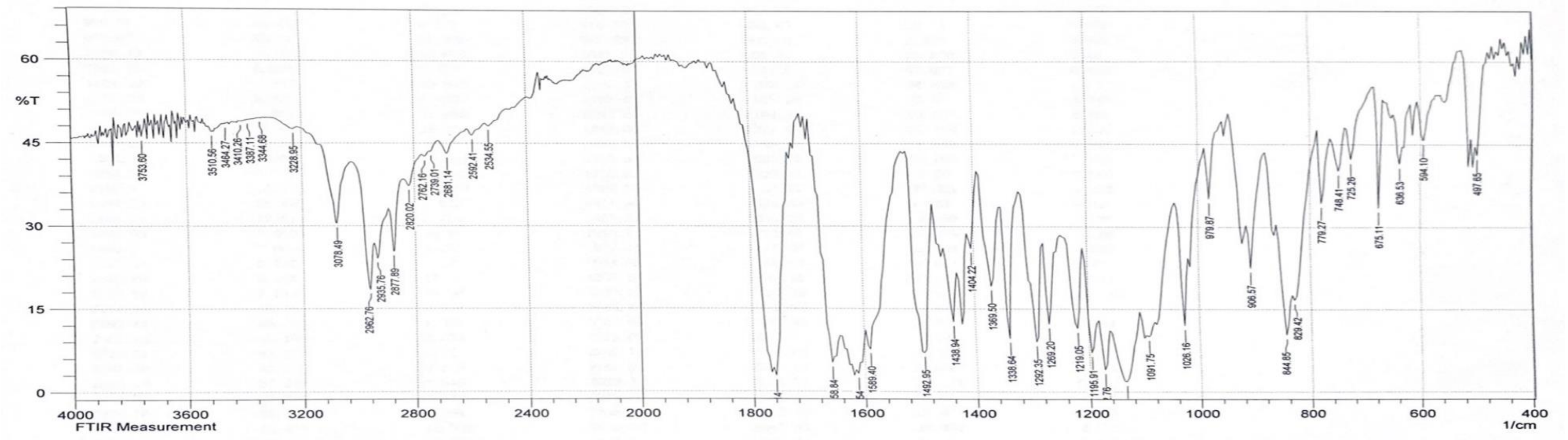

A：7,4’-O-Dimethyl-apigenin

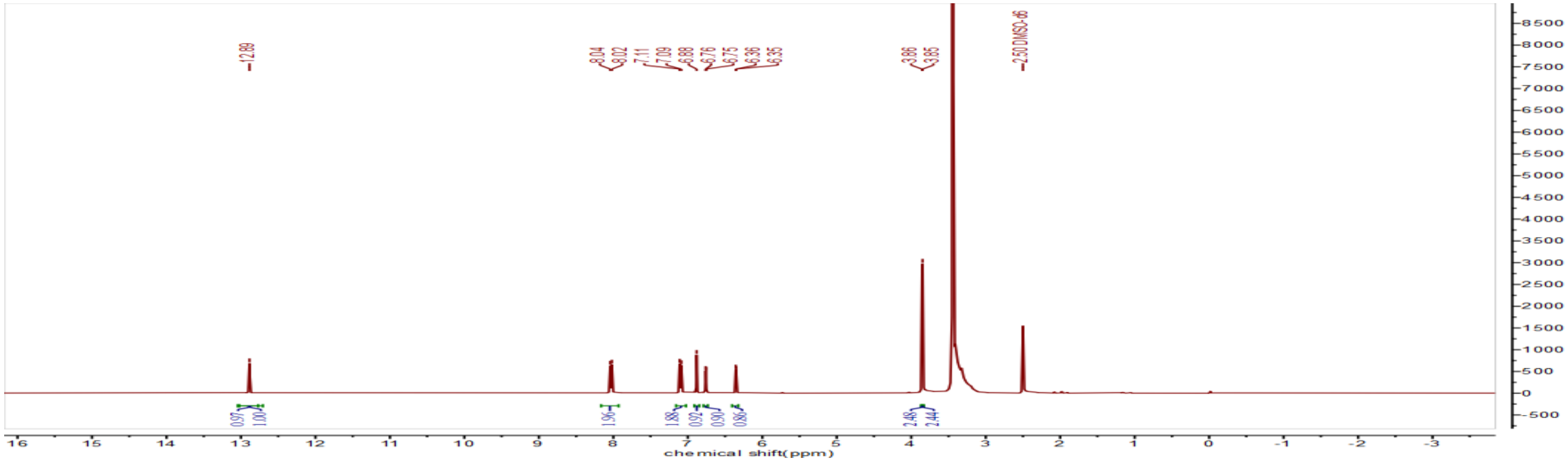

B：7,4’-O-Diethyl-apigenin

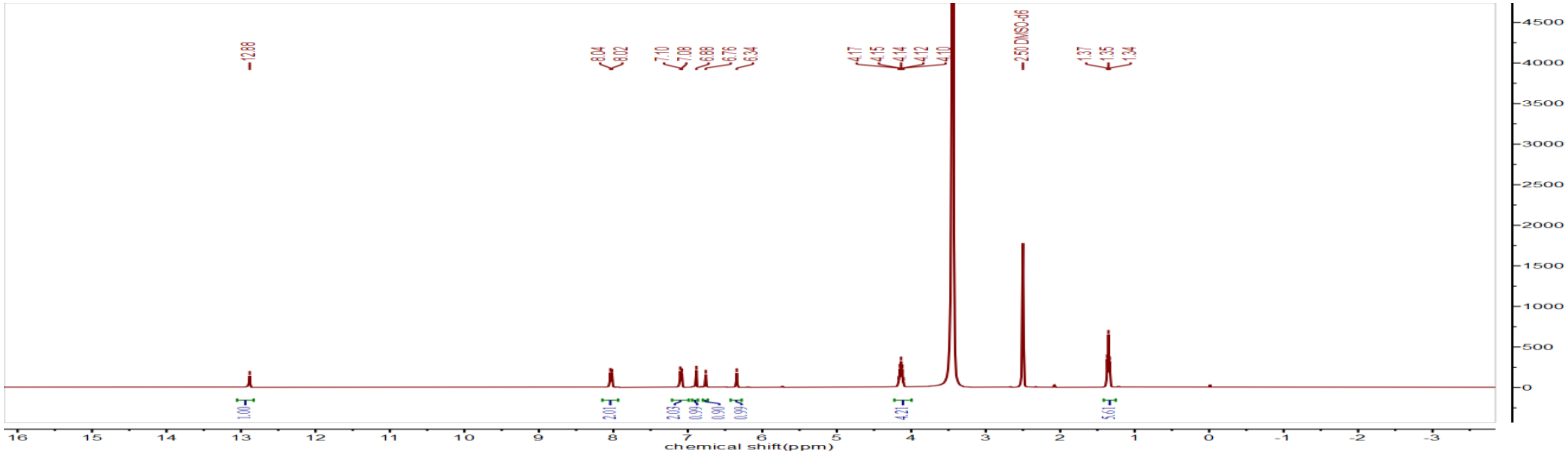

C：5,7,4’-O-Trimethyl-apigenin

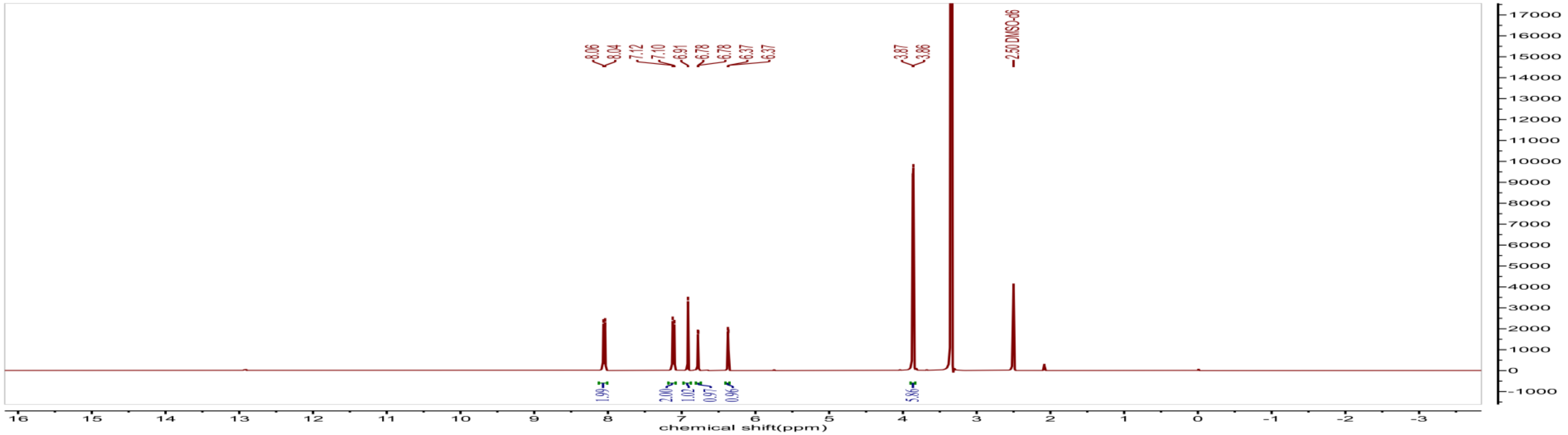

D：5,7,4’-O-Triethyl-apigenin

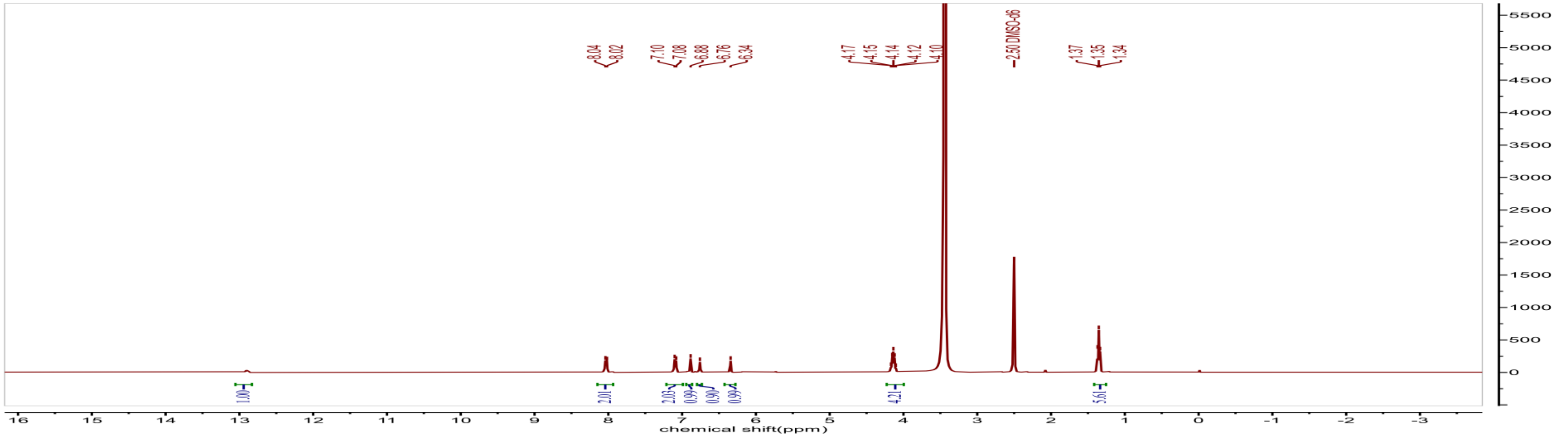

E：7,4’-O-Diacetyl-apigenin

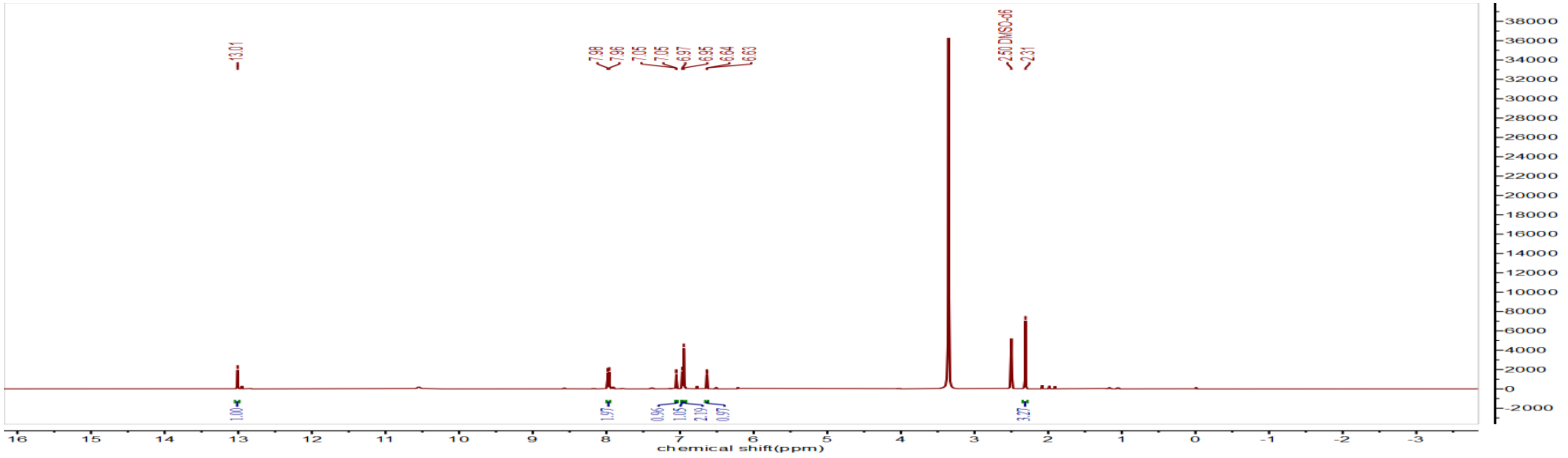

F：7,4’-O-Dipropionyl-apigenin

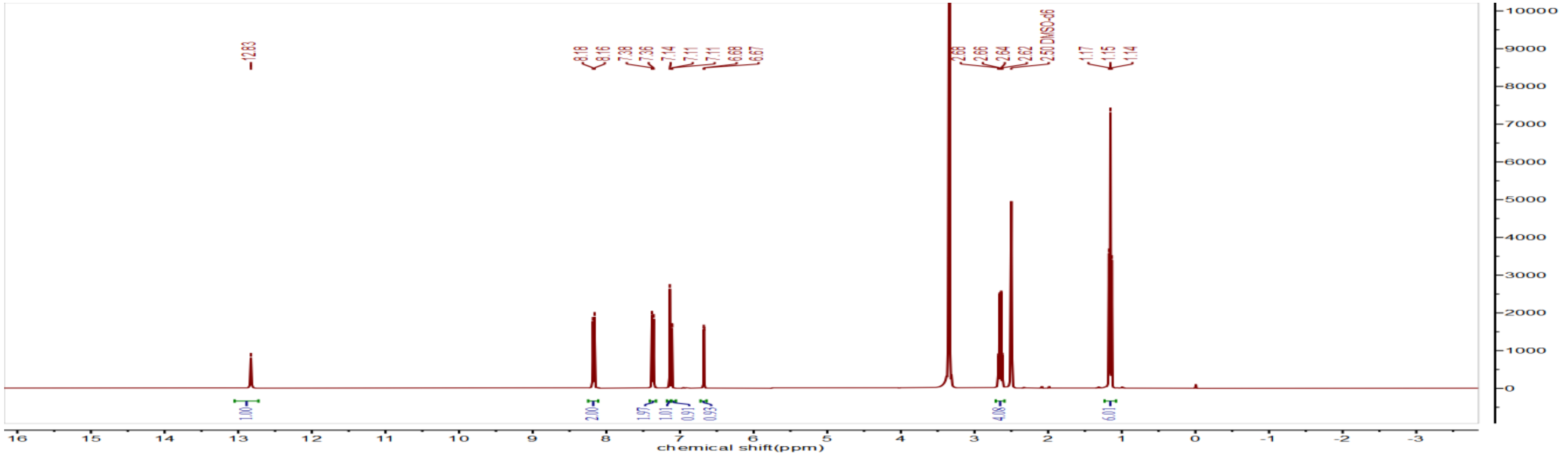

G：7,4’-O-Dibutyryl-apigenin

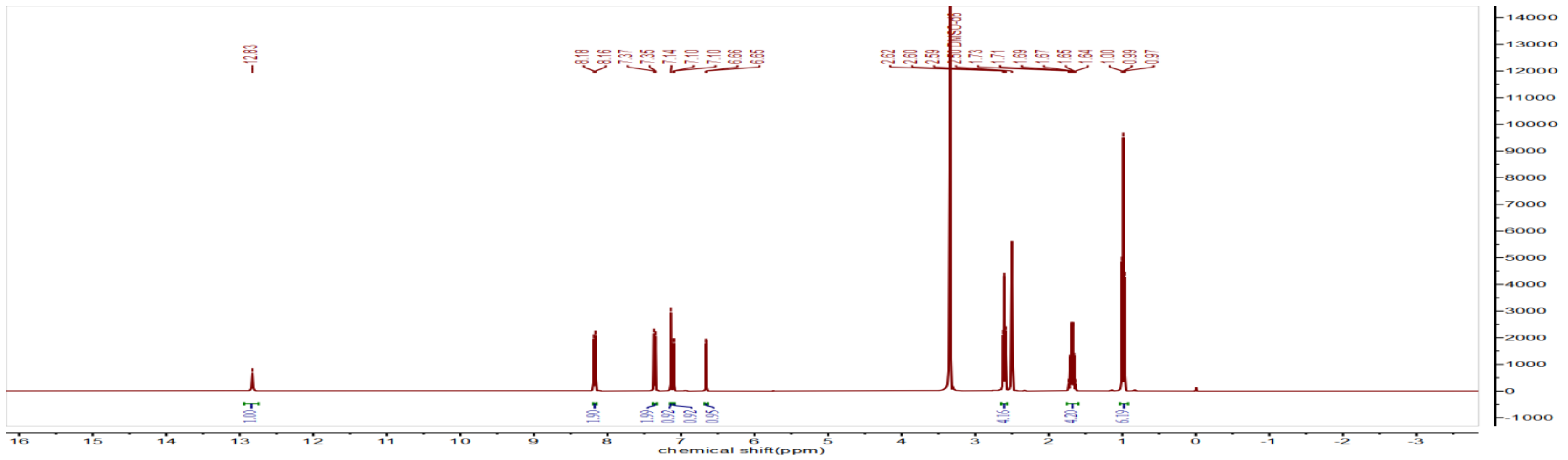

H：5,7,4’ -O-Triacetyl-apigenin

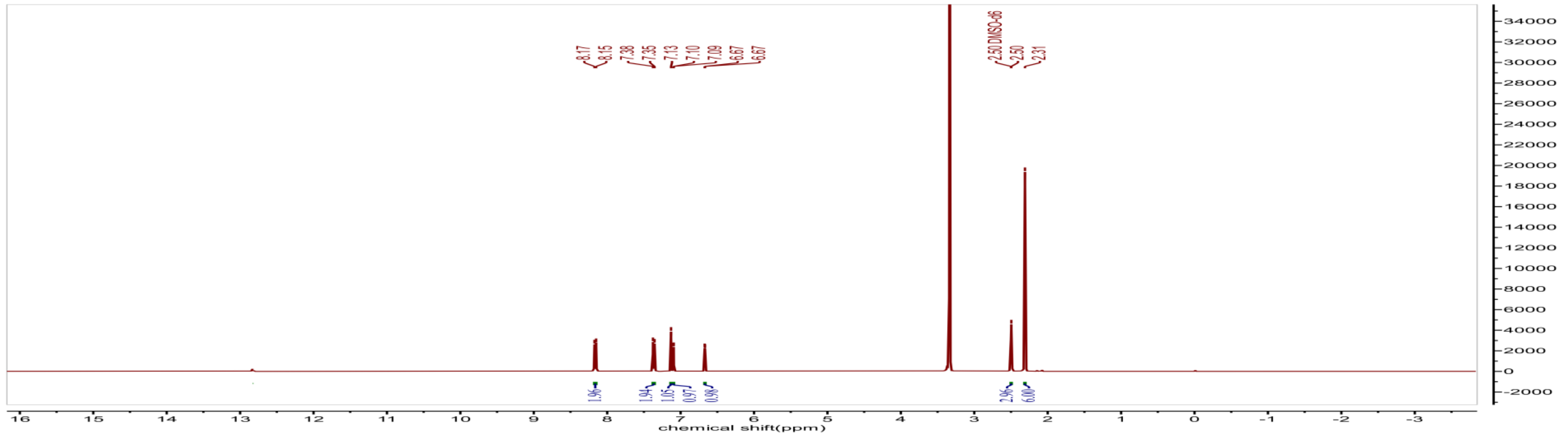

I：5,7,4’-O-Tripropionyl-apigenin

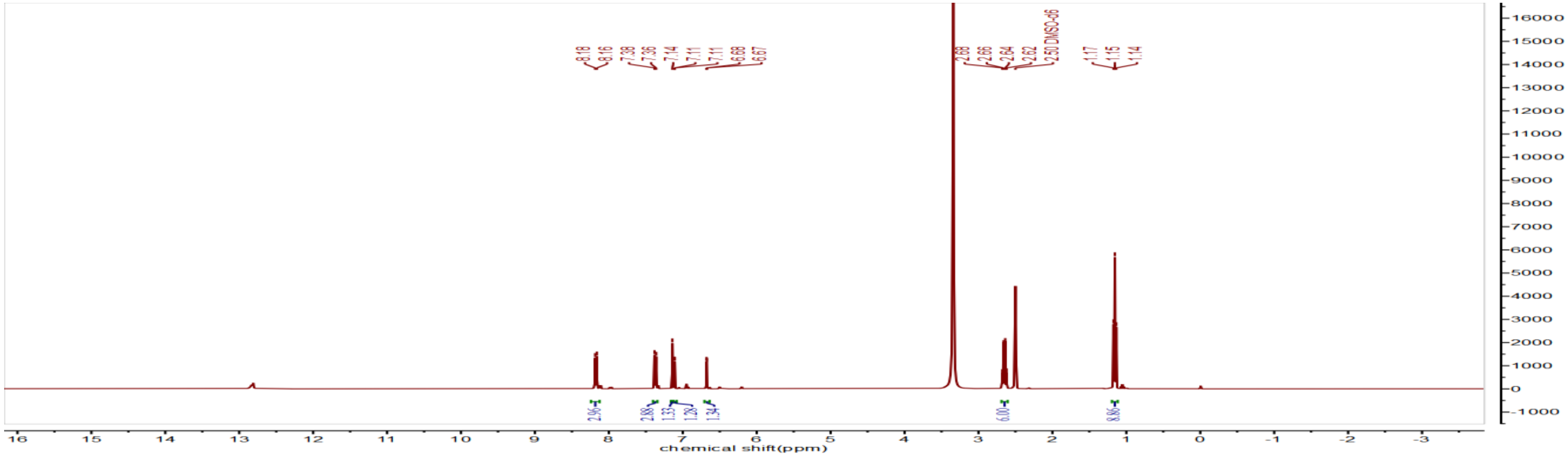

J：5,7,4’-O-Tributyryl-apigenin

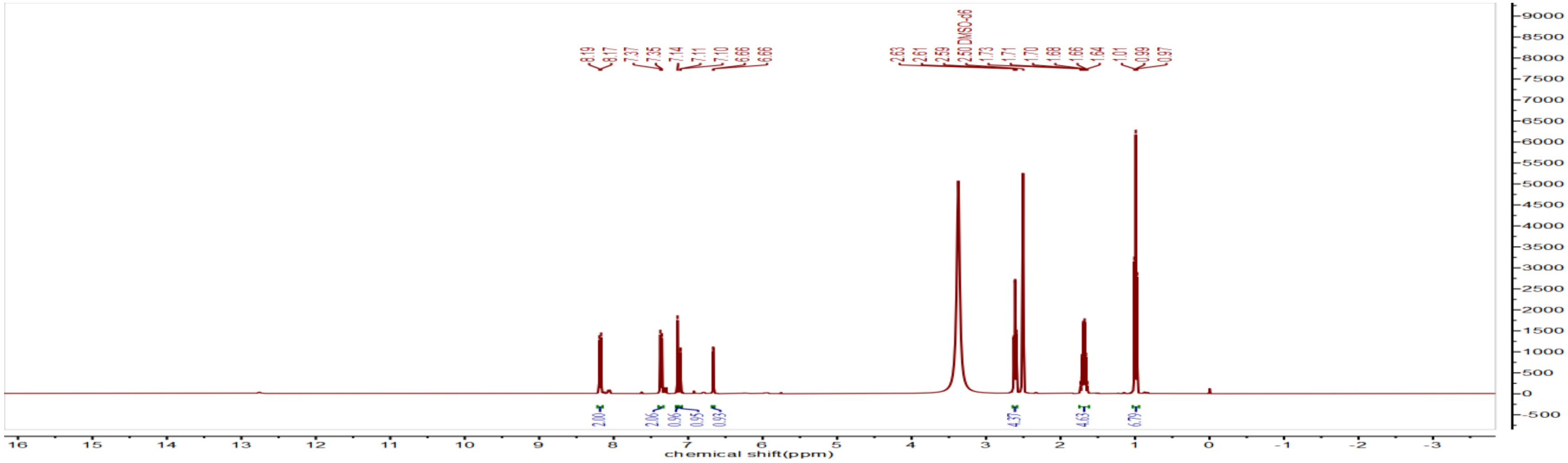

A：7,4’-O-Dimethyl-apigenin

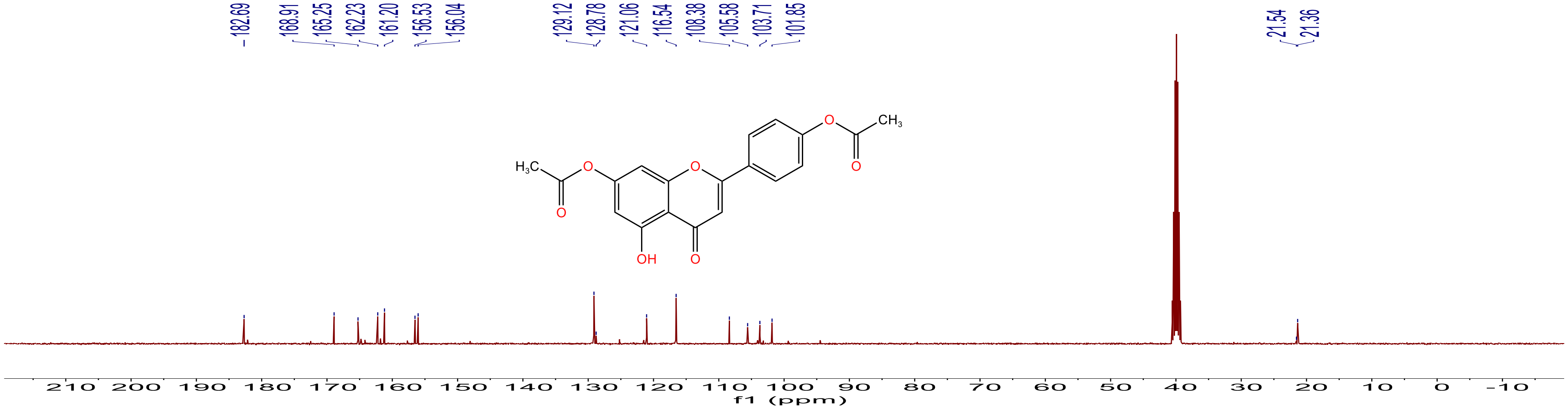

B：7,4’-O-Diethyl-apigenin

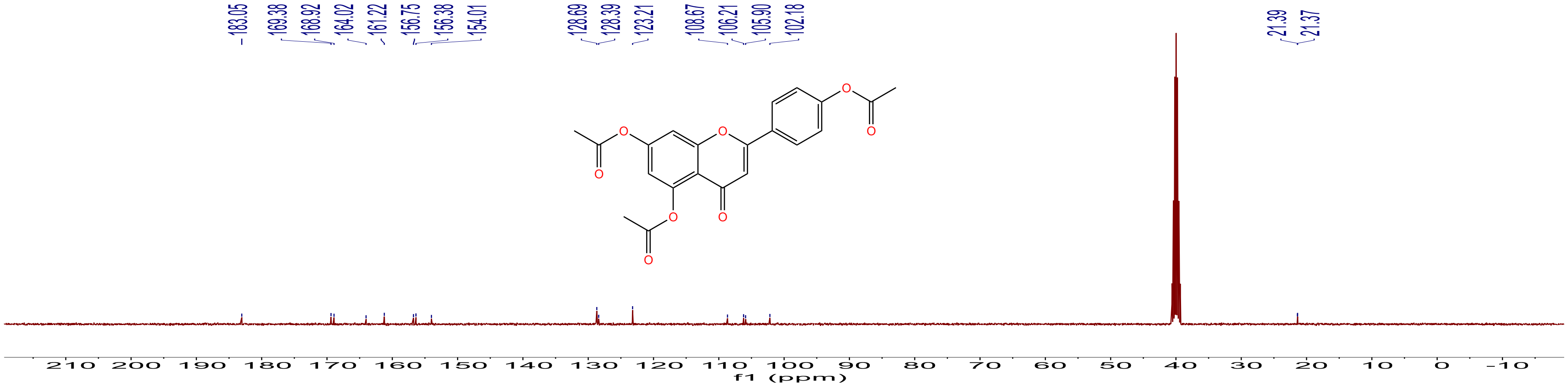

C：5,7,4’-O-Trimethyl-apigenin

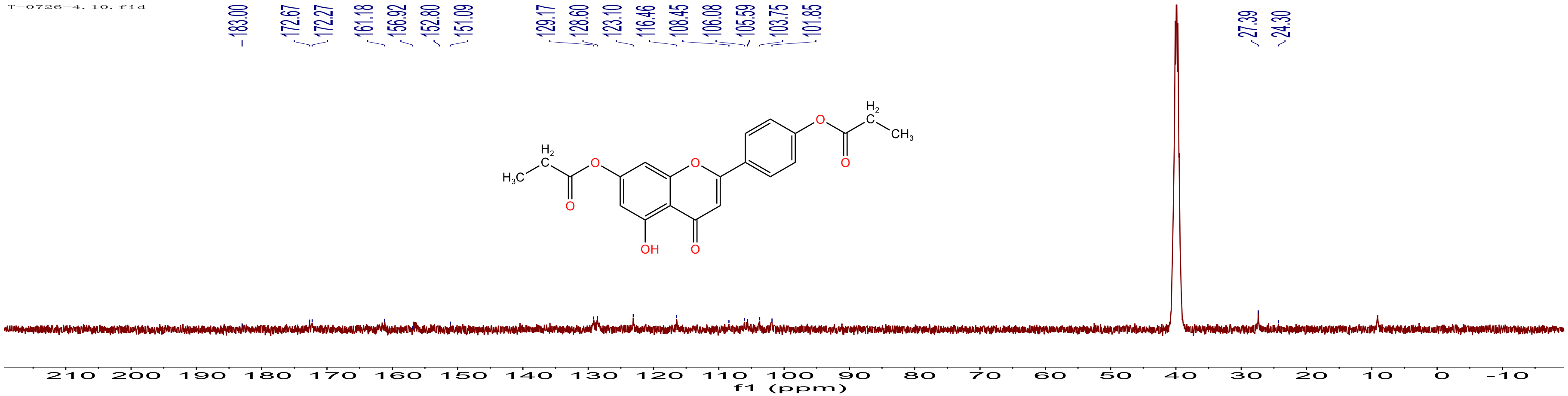

D：5,7,4’-O-Triethyl-apigenin

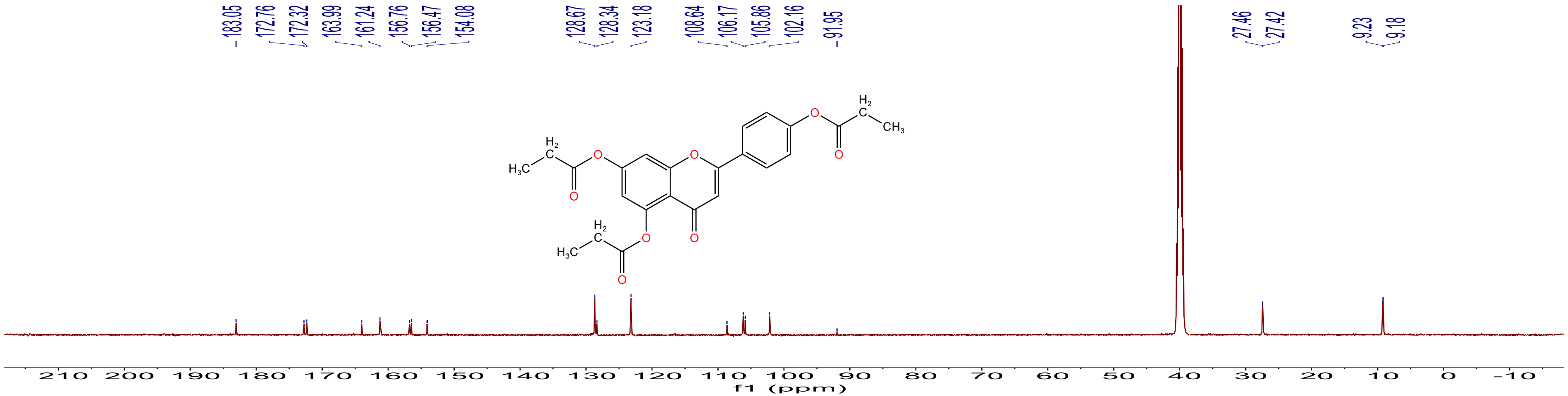

E：7,4’-O-Diacetyl-apigenin

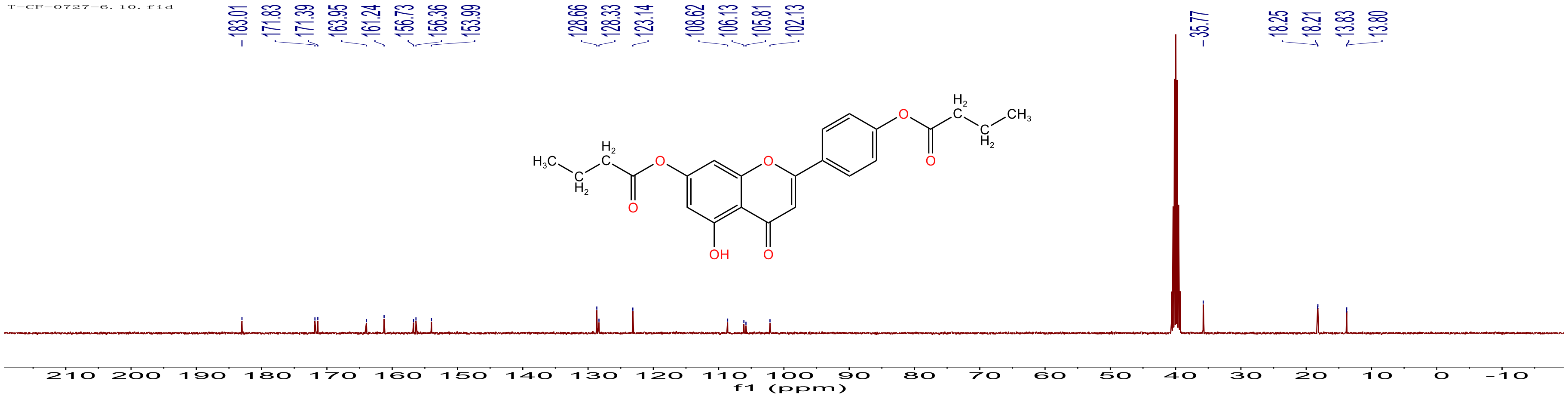

F：7,4’-O-Dipropionyl-apigenin

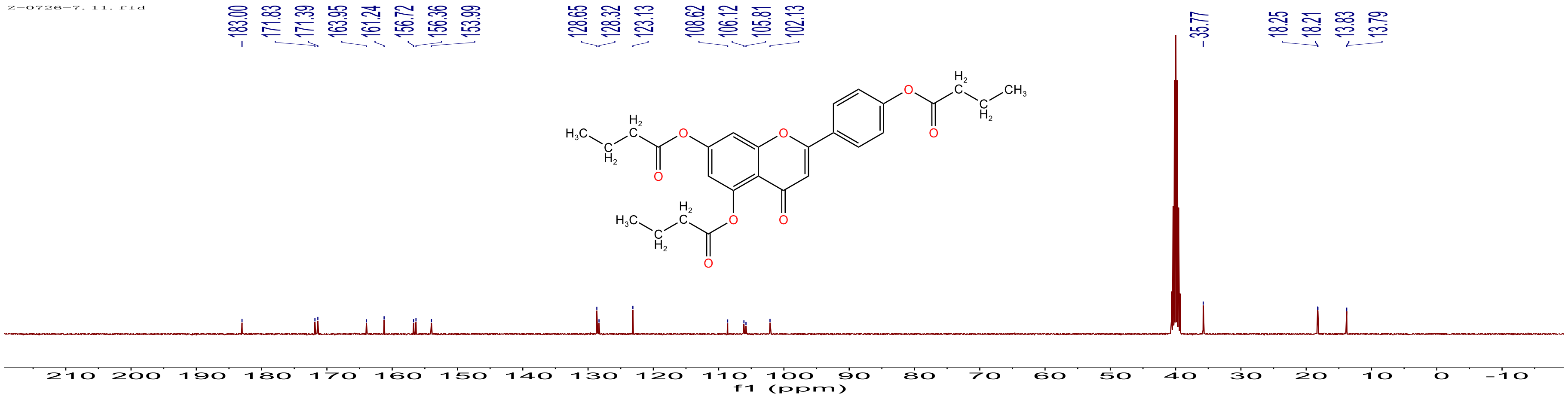

G：7,4’-O-Dibutyryl-apigenin

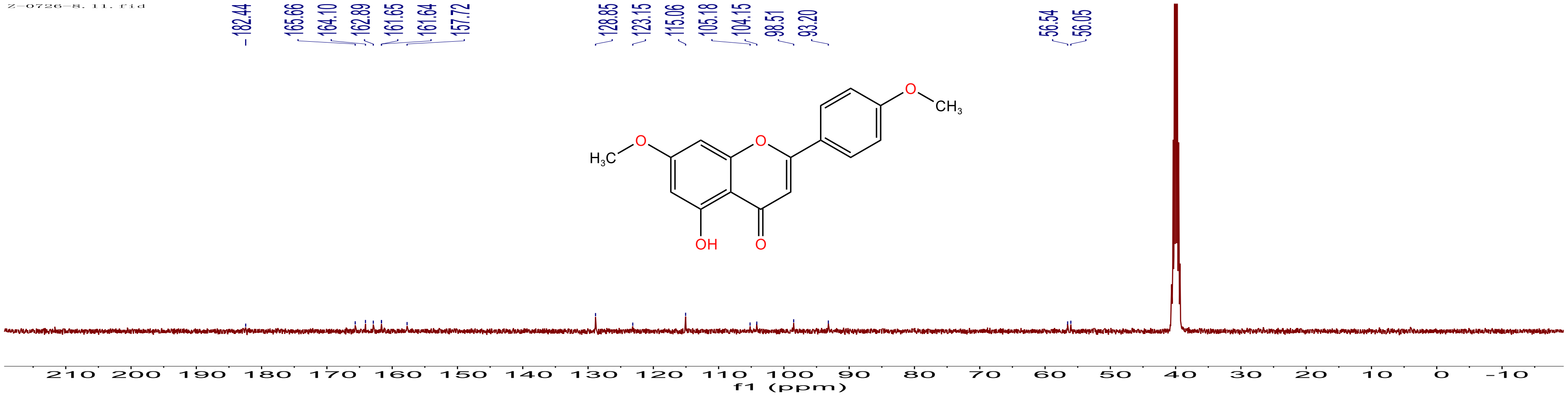

H：5,7,4’ -O-Triacetyl-apigenin

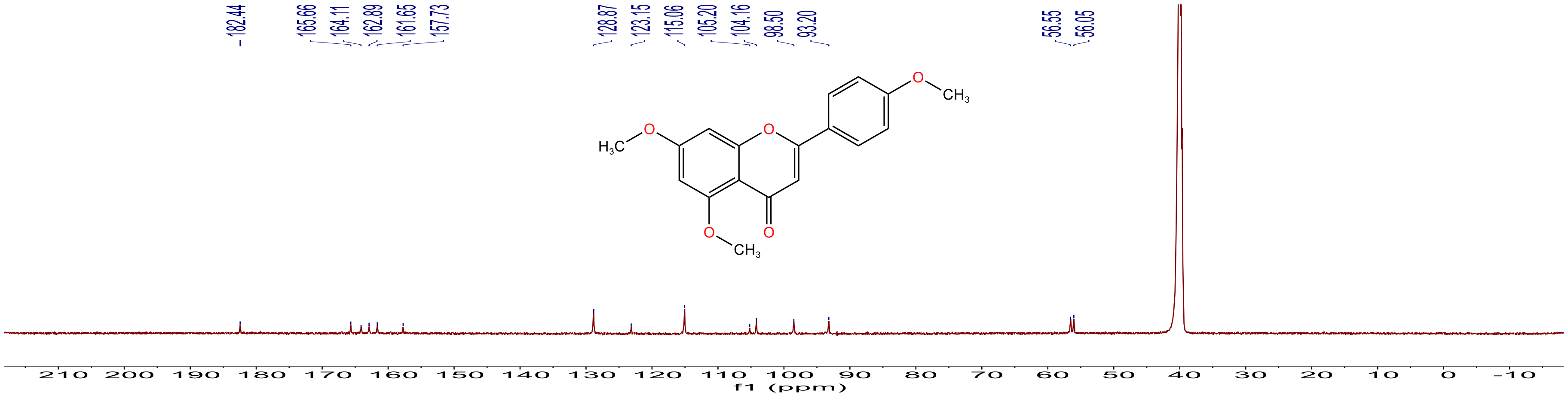

I：5,7,4’-O-Tripropionyl-apigenin

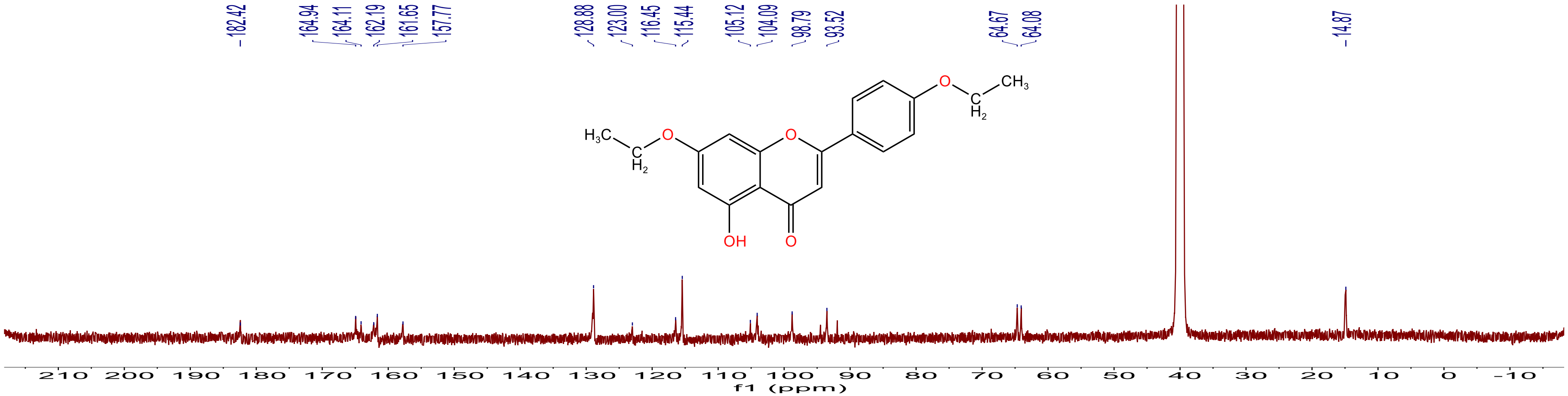

J：5,7,4’-O-Tributyryl-apigenin

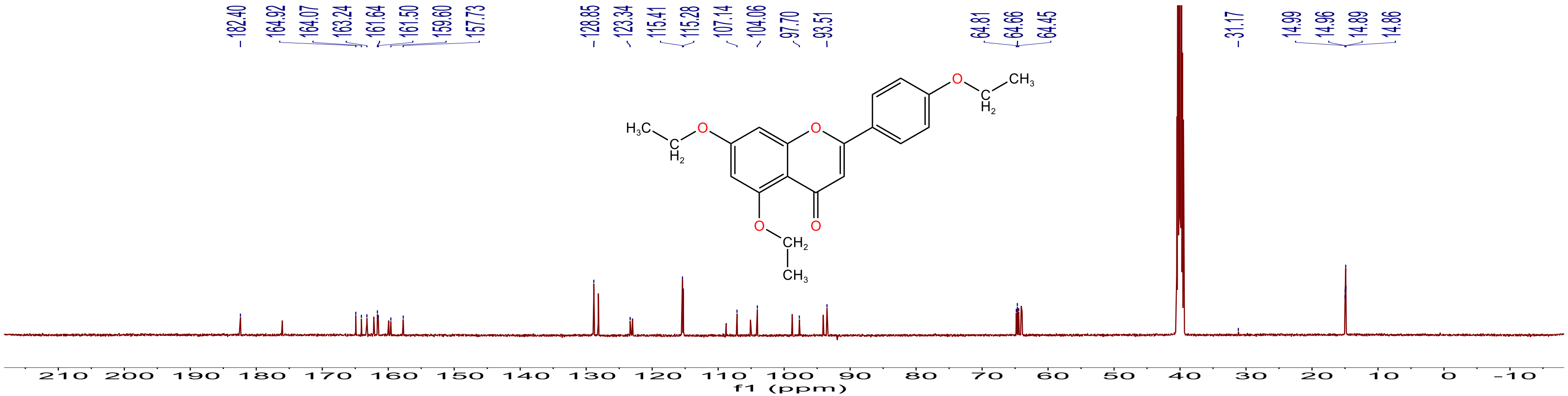

Supplement: Supplementary file 1 [file molecules-31-02768-s001.zip › molecules-4445789-supplementary.pdf]
